# Molecular Steganography Using Multistate Photoswitchable Hydrazones

**DOI:** 10.1021/jacs.5c03668

**Published:** 2025-05-28

**Authors:** Brandon Balamut, Ivan Aprahamian

**Affiliations:** † 6128 Burke Laboratory, Department of Chemistry, 3728 Dartmouth College, Hanover, New Hampshire 03755, United States

## Abstract

The development of photochromic compounds that can have multiaddressable and -stable states is desirable for imparting multistate responses in soft materials. Here, we report on two such photochromes, composed of *para*-NO_2_- and pentafluoro-phenyl functionalized hydrazones connected nonsymmetrically through an isosorbide linker, which exhibit highly efficient, orthogonal, and sequential switching. We took advantage of these properties and the multistability of the four different isomeric states (i.e., *ZZ*, *ZE*, *EZ*, and *EE*) to control the photophysical properties of nematic liquid crystals (LCs). Doping the switches into 5CB, followed by switching to the *EE* state, triggered an unusual cholesteric to focal conic phase transition. We used this property to modulate the opacity of the LC films, resulting in a molecular steganography application.

## Graphical abstract

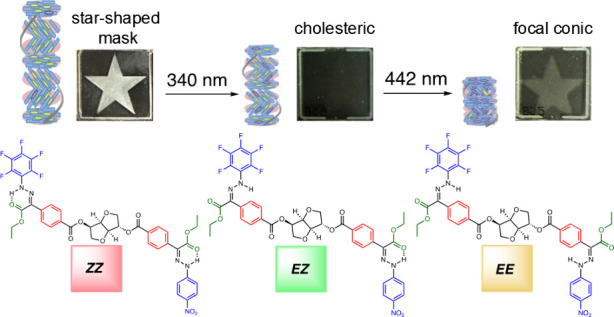

Photochromic molecules^1,2^ are integral building blocks in the advancement of adaptive materials. The light-induced transformations that such systems undergo enable mechanical actuation,^3–6^ the assembly and disassembly of supramolecular superstructures,^7–11^ and the development of various adaptive smart materials.^12–14^ To maximize the utility of these triggers, much effort is spent in tuning their photoswitching properties (i.e., photostationary states (PSSs), quantum yields (Φ), thermal relaxation half-lives (τ_1/2_), and absorption activation wavelengths) as well as other desired characteristics (i.e., high thermal stability and photostability and large geometrical and dipole changes upon isomerization).^15–19^ Another strategy to achieving desirable properties in a switchable system is to covalently link multiple switches with advantageous characteristics^20–23^ resulting in the sequential control over the properties of the system using orthogonal stimuli.^24–29^ For this strategy to work the photoswitchable units need to be electronically decoupled from one another, thus allowing for good spectral separation.^30^ Using this approach numerous combinations of photoswitches composed of idigoids,^31^ fuligmides,^32^ azobenzenes,^33–37^ spiropyrans,^38^ and Stenhouse adducts^39–42^ have been designed. The judicious choice of switches with well separated absorption bands will of course allow for their separate addressability without the need for covalent attachment, though such combinations are few and far between.^21^ The incorporation of different switches in various metal organic^43^ and porous frameworks^44,45^ has recently gained traction as well as a way of taking advantage of the different properties of different switchable systems. Nonetheless, and as with any photochrome, these systems still require further improvements in switching properties (e.g., τ_1/2_, PSSs and photostability).^46^

Hydrazone photoswitches^47^ encompass many desirable features required from photochromic compounds (i.e., thermal half-lives as long as 5300 years,^48^ quantum yields as high as 65%, tunable absorption profiles,^49^ and large changes in geometry upon isomerization, among others^50^). These properties allowed us to incorporate these photoswitches in a plethora of applications including drug delivery,^51^ templated synthesis of γ-cyclodextrin,^52^ modification of the glassy transition state of polymers,^53^ polymer actuation,^54^ manipulation of cholesteric liquid crystals (CLCs),^55–57^ and anion pumping.^58^

Nonetheless, these hydrazones have yet to be combined as part of a multiswitchable system. Here, we report on the development of a multiaddressable chiral switch through the attachment of two different hydrazone photoswitches (Scheme 1) to a chiral isosorbide scaffold. The different photophysical properties of the hydrazones as well as the electronic decoupling between the two photoswitches allows for their orthogonal switching and, hence, fine control over their four-step isomerization sequence (Scheme 2). The chiral nature of the switch, its multiaddressability and sequence switching were used to control the photophysical properties of LC films and induce a unique cholesteric to focal conic phase transition. These properties were used in hiding information in an LC film, which was only retrievable using a specific wavelength of light, i.e., a molecular steganography application.^59–64^

**Scheme 1:**
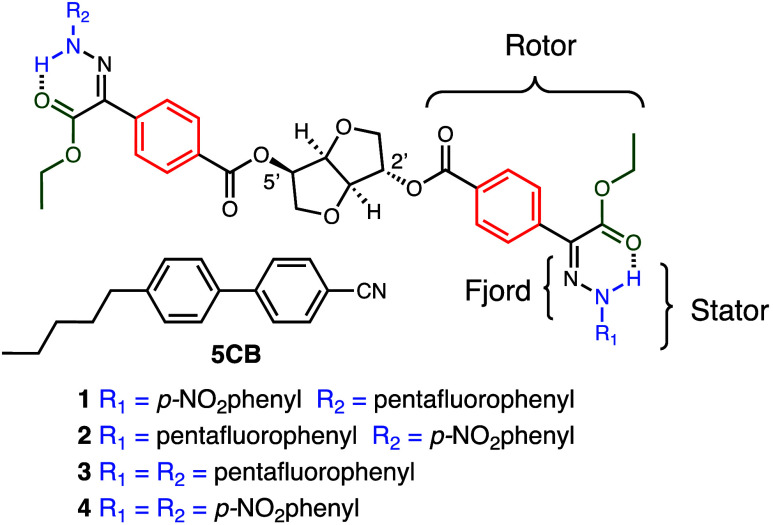
Structures of Chiral Hydrazones **1**–**4** and LC 5CB

**Scheme 2:**
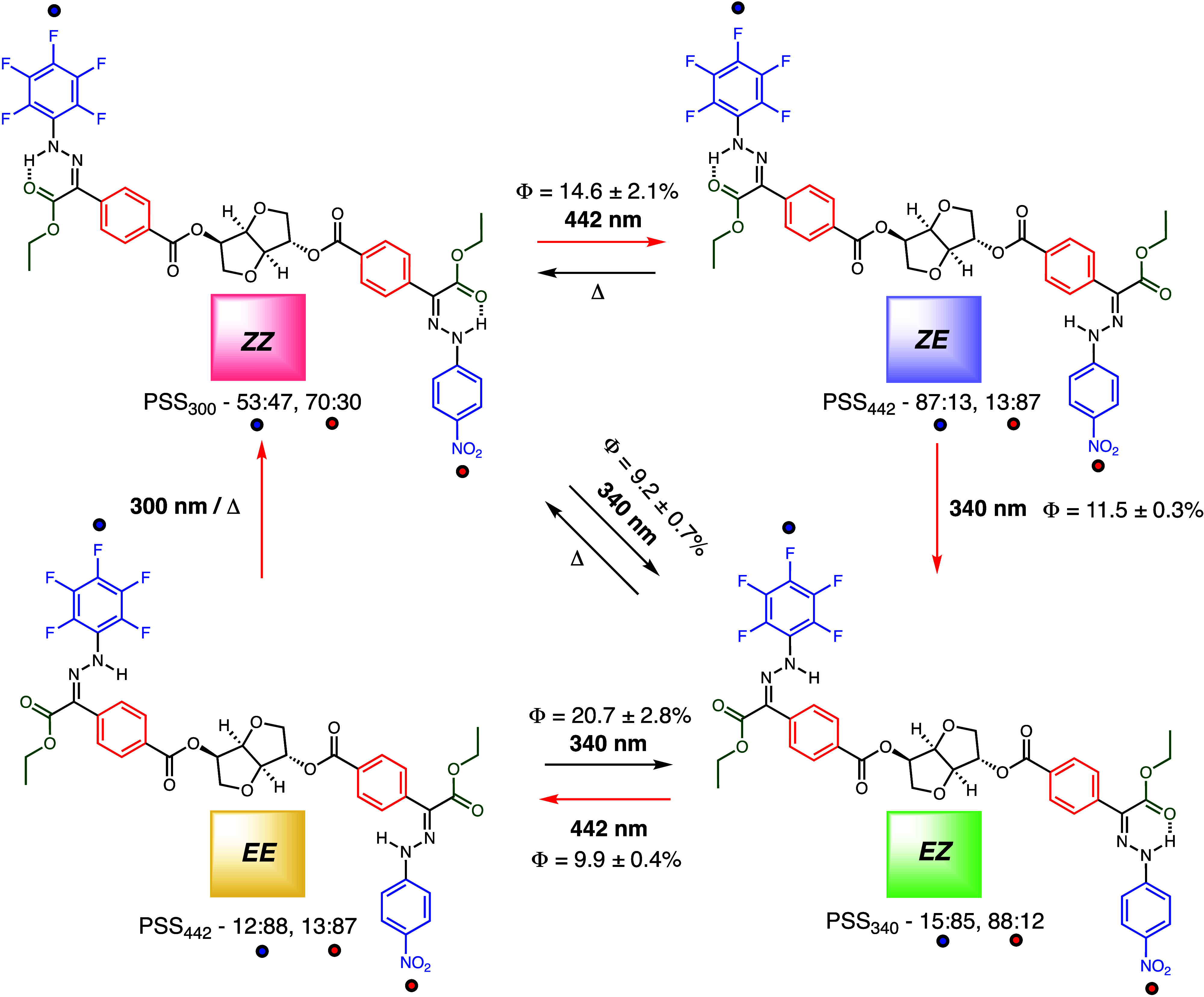
Multistate Isomerization Process of **1**, Showing the Φs and PSSs (of Both the Pentafluoro and *para*-NO_2_ Hydrazone Moieties) for Each Photoisomerization Step^a^

a The red arrows show the sequence specific light-controlled switching cycle.

Compounds **1**–**4** (Scheme 1) were synthesized in a straightforward manner (Schemes S1–S4) with good yields (45–66%) and characterized by NMR spectroscopy and mass spectrometry. The *ZZ* isomer was separated (after column chromatography) as the major configurational isomer in all of the reactions. The assignment was confirmed using the chemical shifts of the intramolecular H-bond between the NH proton and the carbonyl oxygen that resonates at 12.3 and 12.0 ppm for the *para*-NO_2_ and pentafluoro functionalized hydrazones, respectively.

UV–vis, ^1^H NMR and circular dichroism (see Figures S30 and S42 for discussion) spectroscopies were used to study the photophysical and photoisomerization properties of compounds **1–4** (Figures S17–S56 and Tables S1–S3) under aerated conditions. Irradiation of compound **1** (maximum absorption (λ_max_) = 388 nm, absorption coefficient (*ε*) = 46,300 M^–1^ cm^–1^) in acetonitrile with 442 nm light results in a hypsochromic shift (λ_max_ = 364 nm, *ε* = 45,600 M^–1^ cm^–1^) indicating that *ZZ* → *ZE* isomerization has taken place. The ^1^H NMR spectra (Figures 1a and b) show the NH signal of the *para*-NO_2_ portion of the switch shifting from 12.3 to 9.0 ppm confirming this conclusion. This process results in the conversion of 87% of *para*-NO_2_ to its *E* form at PSS_442_ with an overall Φ of 14.6 ± 2.1%. The ^1^H NMR spectrum (i.e., appearance of a NH signal at 8.0 ppm) also shows that 13% of the pentafluoro switch is converted to its *E* form with 442 nm light irradiation. We speculate that the excitation of the *para*-NO_2_ portion of the switch causes a slight bathochromic shift in the absorbance of the pentafluoro portion, thus allowing partial isomerization with 442 nm light.^65^ Subsequent irradiation with 340 nm light results in a bathochromic shift (λ_max_ = 392 nm, *ε* = 44,500 M^–1^ cm^–1^), which based on the ^1^H NMR spectrum (Figure 1c) results from the *ZE* → *EZ* isomerization process; i.e., the NH signals of the pentafluoro and *para*-NO_2_ portions shift from 12.0 to 8.0 ppm and 9.0 to 12.3, respectively. The process results in the conversion of 85% of the pentafluoro half to its *E* form and 88% of the *para*-NO_2_ half to its *Z* configuration at PSS_340_ with Φ of 11.5 ± 0.3%. Alternatively, irradiation of the pristine state with 340 nm light results in *ZZ* → *EZ* isomerization: the ^1^H NMR spectrum (Figure S24) shows the NH signal of the pentafluoro portion of the switch shifting from 12.0 to 8.0 ppm. The process results in the conversion of 88% of the pentafluoro half to its *E* form and 89% to the *para*-NO_2_ half to the *Z* form at PSS_340_ with Φ 9.2 ± 0.7%. Further irradiation with 442 nm light results in the *EZ* → *EE* process which is accompanied by a hypsochromic shift (λ_max_ = 380 nm, *ε* = 38,800 M^–1^ cm^–1^) and the NH signal of the *para*-NO_2_ portion of the switch shifting from 12.3 to 9.0 ppm (Figure 1d). This process results in the conversion of 88% of the pentafluoro and 87% of the *para*-NO_2_ halves to their *E* forms at PSS_442_ with Φ 9.9 ± 0.4%. Irradiation with 340 nm light reverses the process (PSS_340_ of 87% of the pentafluoro *E* and 88% of the *para*-NO_2_ *Z* forms; Φ = 20.7 ± 2.8%). This switching cycle can be repeated multiple times (Figures S23 and S37), indicating that this part of the process is photofatigue resistant. Finally, irradiation of the *EE* state with 300 nm light results in partial restoration of the *ZZ* state (PSS_300_ of 53% of the pentafluoro *Z* and 70% of the *para*-NO_2_ *Z* forms; see Scheme 2 and Figure S28 for more details). As expected, the use of the relatively high energy light source causes some photodegradation upon photoswitching, and thus Φ was not measured. Interestingly, and because of the bistability and addressability of the hydrazones, the photoswitching process results in a sequence specific switching cycle, i.e., *ZZ → ZE → EZ → EE → ZZ* (highlighted with red arrows in Scheme 2).

**Figure 1:**
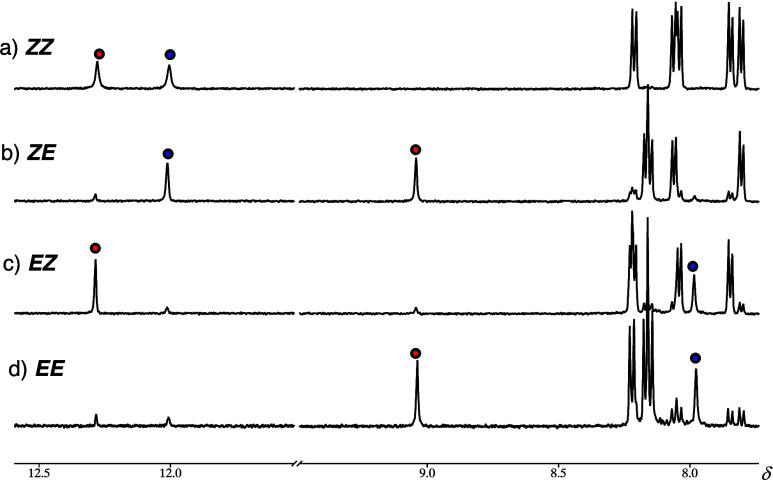
^1^H NMR spectra of the a) pristine, b) PSS_442_, c) PSS_340_, and d) PSS_442_ of compound **1** in CD_3_CN at 294 K. The red and blue dots indicate the NH proton signals for the *para*-NO_2_ and pentafluoro functionalized hydrazones, respectively.

The diastereomeric counterpart **2** undergoes a similar photoswitching process, and the photophysical data can be found in the Supporting Information (Figures S31–S45 and S51–S55 and Table S2). The τ_1/2_ measurements for the *EE* → *ZZ* process in **1** and **2** showed a nonsymmetric thermal process (Table S4) with the pentafluoro portion relaxing faster than the *para*-NO_2_ (147 ± 8 vs 41 ± 4 years in **1** and 138 ± 13 vs 35 ± 13 years in **2**). The symmetric control compounds **3** and **4** undergo similar processes (Figure S56 and Tables S3 and S4).

To take advantage of the multistep switching of compounds **1** and **2** and their chiral nature, we decided to use them as photoswitchable chiral dopants that can be used in modulating the properties of LCs.^66–72^ The helical twisting power (β, i.e., the ability of the dopant to transfer chiral information on the LC) values for **1**–**4** (<1 mol % doping) and their photoswitched isomers in the LC 5CB (Scheme 1) were determined using the Grandjean-Cano wedge method (Table S5 and Figures S57–S59).^56,73^ In all cases except for **3** (Figure S59) the value could not be measured for the pristine (*ZZ*) compound because the β value was too small. Irradiating compound **1** with 442 nm light results in a β value of 53 μm^–1^ (*ZE* isomer), which decreases to 48 μm^–1^ upon 340 nm light irradiation (*EZ* isomer), followed by an increase to 110 μm^–1^ after another round of 442 nm light irradiation (*EE* isomer). For compound **2** the sequence of irradiation first decreases the β value from 62 to 35 μm^–1^ and then increases to 81 μm^–1^. The slight differences between **1** and **2** originate from the different substitution pattern of the hydrazones at the 2′ and 5′ positions of the isosorbide unit, as shown previously by us (i.e., larger changes in β values are observed in *para-*NO_2_ functionalized hydrazones when the rotor phenyl group is connected to the 5′ position of isosorbide, whereas connection at the 2′ position results in minimal changes in the β value upon isomerization). Small amounts of *Z* isomer of the *para-*NO_2_ functionalized hydrazone connected at the 5′ position result in drastic lowering of the β value.^56^

Next, we prepared adaptive reflective films, by doping **1** or **2** (>2.5 mol %) into 5CB. The transmittance of light from the films was measured after sequential irradiation with 442, 340, and 442 nm light resulting in the modulation of the reflectance of 650–1100 nm light from the surface. Interestingly the *EE* isomer of **1** and **2** in 5CB results in an unusual transition from a cholesteric to a focal conic texture. While we have previously observed a cholesteric to a smectic A* transition using an isosorbide-based switchable dopant,^55^ this is the first we encountered a light-induced alignment change. We speculate that this phenomenon arises from frustrations that occur in the LC films^74^ and is dependent on the pentafluoro portion of the switch; i.e., compound **3** does not show any evidence of a cholesteric phase at increased concentrations but rather exhibits a focal conic texture (Figure S60), whereas compound **4** only behaves as cholesteric. LC films with equimolar concentrations of **3** and **4** do not afford cholesteric textures, thus showcasing the benefit of the covalent attachment of the two different hydrazones (Figure S61). Considering that the focal conic phase scatters light across the visible to NIR of the electromagnetic spectrum instead of reflecting visible light (Figures S62–S67), i.e., effectively blocking 75% of light transmittance through the LC film, we decided to use the focal conic phase in a photoprogammable steganography application (Figure 2).^59,64^

**Figure 2:**
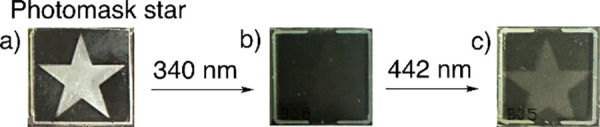
An LC film of **1** and 5CB (a) having a star-shaped photomask, which upon irradiation with 340 nm light for 30 s results in the image in (b) where no information can be seen, even though a *ZZ* → *EZ* isomerization occurred in the star-shaped region. Upon further irradiation with 442 nm light for 90 s (c), the written information is revealed, where the opaque star shape results from the *EE* state and the black region results from the mixture of *ZZ* and *ZE* states. The molar photon flux values of the light source at 340 and 442 nm are 7.76 × 10^–8^ and 6.56 × 10^–8^ mol/s, respectively.

We used a star-shaped mask (Figure 2a) followed by irradiation with 340 nm light, resulting in a *ZZ* to *EZ* conversion in the unmasked area, to imprint the image of a star onto the LC film. While the information is written on the surface (Figure 2b), it is not apparent because the LC is reflecting in the NIR region. Next, we irradiated the whole LC film without the photomask with 442 nm light, thus converting the *EZ* isomer into the *EE* one, i.e., resulting in a change from the cholesteric phase to a focal conic texture, revealing the hidden image (Figure 2c).

In conclusion, two new nonsymmetric bis-hydrazones have been developed and their orthogonal photoswitching elaborated. We took advantage of their multiaddressability to control the photophysical properties of nematic LCs, and the photoactivated cholesteric to focal conic phase transition enabled us to use the LC in a steganography application. Although stimuli-responsive LCs^75–77^ and multiresponsive photochromic systems^78–80^ have been separately used in steganography, our approach combines these two areas and results in a straightforward and reusable system, with a naked-eye readout.

## Supplementary Material



## Notes

General methods, experimental procedures, NMR spectra of key compounds, photoisomerization studies, kinetic studies, and details about doping experiments (PDF)
