# Supplementary material for: Molecular Steganography Using Multistate Photoswitchable Hydrazones
Source: J Am Chem Soc. 2025 May 28;147(23):19444–9. doi: 10.1021/jacs.5c03668 (PMC12164268; doi:10.1021/jacs.5c03668)
Supplement: Supplementary file 1 [file ja5c03668_si_001.pdf]

Supporting Information

Molecular Steganography Using Multistate Photoswitchable Hydrazones

Brandon Balamut, Ivan Aprahamian*

Department of Chemistry, Dartmouth College, Hanover, NH 03755

Email: ivan.aprahamian@dartmouth.edu

Table of Contents

1. **General Methods** **S3**
2. **Synthesis and Characterization of Compounds** **S4**
3. **NMR Characterization** **S10**
4. **Photoisomerization Studies** **S18**
5. **Determination of the Quantum Yield** **S33**
6. **Determination of the Thermal Isomerization Half-Life** **S42**
7. **HTP (β) and Reflectance of Adaptive Films** **S44**
8. **References** **S51**

1. General Methods

All reagents and starting materials were obtained from commercial sources and used without further purification unless otherwise noted. All reactions were done under normal atmosphere unless otherwise noted. Compounds were purified by column chromatography using silica gel (SiliCycle®, 60 Å, 230-400 mesh) as the stationary phase and eluting solvents are reported as ratios unless otherwise noted. Recrystallizations were performed with HPLC grade solvents. Deuterated solvents were obtained from Cambridge Isotope Labs and used without further purification. ^{1}H and ^{13}C NMR spectra were recorded on 500 or 600 MHz instruments with working frequencies of 500.13 and 600.13 MHz for ^{1}H nuclei and 125.8 or 150.9 MHz for ^{13}C nuclei, respectively. Chemical shifts are quoted in ppm relative to tetramethylsilane (TMS), using the residual solvent peak as the reference standard. ESI mass spectra were obtained on a Shimadzu LCMS-8030 mass spectrometer. UV-Vis and transmittance spectra were recorded on a Shimadzu UV-1800 UV-Vis spectrophotometer.

Irradiation experiments were conducted with a stand-alone xenon arc lamp system (Model:LB-LS/30, Sutter Instrument Co.), outfitted with a SMART SHUTTER controller (Model: LB10-B/IQ, Sutter Instrument Co.) and a liquid light guide LLG/250. 340 (part number: 340HC10-25) and 442 (part number: 442FSX10-25) nm light filters, purchased from Andover Corporation, were used in the irradiation experiments.

The textures of the liquid crystal samples were evaluated using an Olympus BX53 polarized optical microscope, the photomicrographs were captured using an INSTEC MITO2-MC camera. The planar and homeotropically aligned liquid crystal cells were purchased from INSTEC, and the wedge cells were purchased from EHC Co Ltd. Liquid crystalline host 5CB was purchased from Ambeed.

2. Synthesis and Characterization of Compounds

Scheme S1. Synthesis of hydrazones **6** and **7** with carboxylic acid substitution at the *para*– position of the benzoyl ester unit.

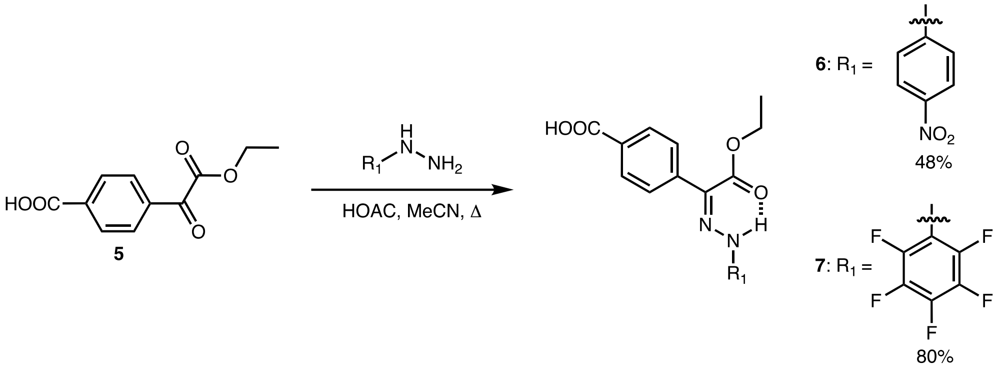

5: This compound was synthesized by following a reported procedure.[S1] Compound **5** was obtained in 90% yield (0.5 g) and its identity confirmed by comparing its ^{1}H NMR spectrum with the reported one; ^{1}H NMR (500 MHz, DMSO) δ 13.52 (s, 1H), 8.16 – 8.06 (m, 4H), 4.44 (q, *J* = 7.1 Hz, 2H), 1.35 (t, *J* = 7.1 Hz, 3H).

6: 4-nitrophenylhydrazine (0.147 g, 0.774 mmol) and a catalytic amount of acetic acid (HOAc) were added to a MeCN solution of compound **5** (0.154 g, 0.693 mmol) under N_2 atmosphere. The resulting mixture was refluxed overnight and then cooled to room temperature. MeCN was removed under vacuum and the crude residue was dissolved in methylene chloride, washed twice with water and once with brine, and dried over Na_2SO_4. The solvent was removed under reduced pressure and the residue was subjected to silica gel column chromatography (4:6 EtOAc:Hexane) followed by recrystallization to afford 160 mg of **6** (48% yield) as a yellow solid. m.p. 205–206 °C; ^{1}H NMR (600 MHz, DMSO) δ 11.73 (s, 1H), 8.22 (d, *J* = 9.1 Hz, 2H), 7.99 (d, *J* = 8.4 Hz, 2H), 7.80 (d, *J* = 8.3 Hz, 2H), 7.56 – 7.51 (m, 2H), 4.43 (q, *J* = 7.1 Hz, 2H), 1.33 (t, *J* = 7.1 Hz, 3H); ^{13}C NMR (151 MHz, DMSO) δ 167.43, 162.82, 149.70, 141.39, 138.93, 134.87, 129.87, 129.82, 127.78, 126.25, 114.24, 62.46, 14.29; *m/z* found [M^-] for $C_{17}H_{14}N_3O_6$ 356.0884 (calcd. 356.0883)

7: Pentafluorophenylhydrazine (0.210 g, 1.06 mmol) and a catalytic amount of HOAc were added to a MeCN solution of compound **5** (0.223 g, 1.00 mmol) under N_2 atmosphere. The resulting mixture was refluxed overnight and then cooled to room temperature. MeCN was removed under

vacuum and the crude residue was dissolved in methylene chloride, washed twice with water and once with brine, and dried over Na_2SO_4. The solvent was removed under reduced pressure and the residue was subjected to silica gel column chromatography (3:7 EtOAc:Hexane) followed by recrystallization to afford 0.320 mg of **7** (yield 80%) as an orange-brown solid. m.p. 220–221 °C; ^{1}H NMR (500 MHz, DMSO) δ 11.55 (s, 1H), 7.95 (d, *J* = 8.3 Hz, 2H), 7.71 (d, *J* = 8.3 Hz, 2H), 4.38 (q, *J* = 7.1 Hz, 2H), 1.31 (t, *J* = 7.1 Hz, 3H); ^{13}C NMR (151 MHz, DMSO) δ 167.00, 166.71, 162.17, 138.85, 134.49, 133.27, 130.41, 129.89, 129.48, 129.13, 127.82, 61.93, 13.79. *m/z* found [M$^-$] for $C_{17}H_{10}N_2O_4F_{10}$ 401.0559 (calcd. 401.0561)

Scheme S2. Synthesis of compounds **8** and **9** with isosorbide substitution at the *para*– position of the benzoyl ester unit.

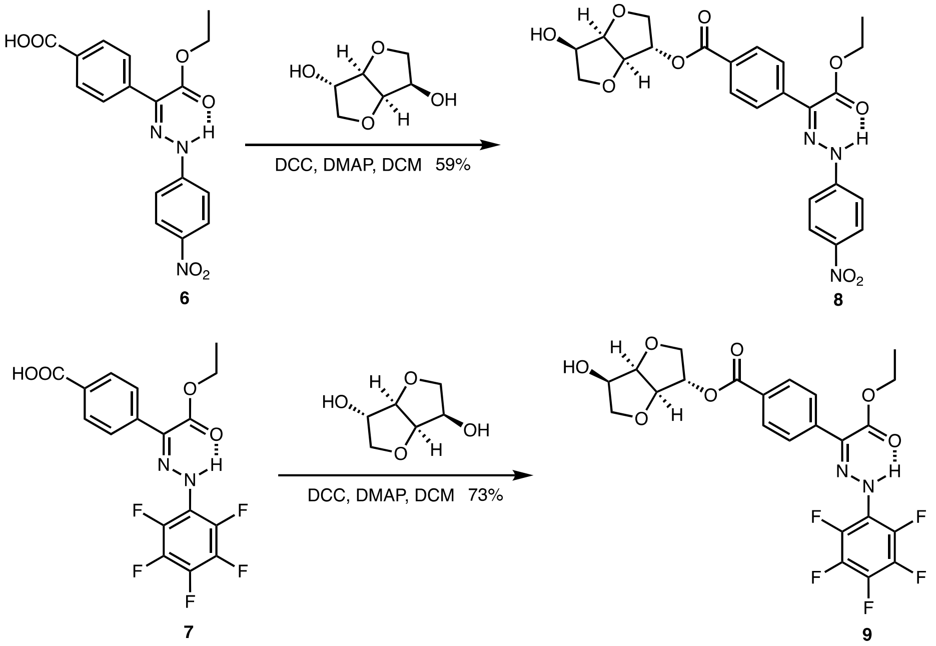

8: A mixture of compound **6** (0.010 g, 0.2 mmol), DCC (0.062 g, 0.300 mmol), and DMAP (0.002 g) were mixed in 7 mL of dry methylene chloride at 0° C. After 20 minutes, excess *d*-isosorbide (0.164 g, 1.12 mmol) was added and the solution stirred for 12 hours. The crude mixture was extracted with methylene chloride and washed twice with water and once with brine. The mixture was subjected to column chromatography (4:6 EtOAc:Hexane) yielding 80 mg (59% yield) of compound **8** as a yellow solid. m.p. 88–89 °C; ^{1}H NMR (600 MHz, CD_2Cl_2) δ 12.63 (s, 1H), 8.23 (d, *J* = 9.1 Hz, 2H), 8.08 – 8.03 (m, 2H), 7.82 – 7.77 (m, 2H), 7.40 – 7.35 (m, 2H), 5.45 (d, *J* = 3.6 Hz, 1H), 4.72 (t, *J* = 4.9 Hz, 1H), 4.63 (d, *J* = 4.5 Hz, 1H), 4.41 (q, *J* = 7.1 Hz, 2H), 4.36 – 4.29

(m, 1H), 4.19 (d, J = 10.8 Hz, 1H), 4.11 (dd, J = 10.8, 3.7 Hz, 1H), 3.91 (dd, J = 9.4, 6.0 Hz, 1H), 3.59 (dd, J = 9.4, 5.9 Hz, 1H), 2.57 (d, J = 7.4 Hz, 1H), 1.37 (t, J = 7.2 Hz, 3H). ^{13}C NMR (151 MHz, CD_2Cl_2) δ 165.56, 163.45, 148.55, 140.78, 129.71, 129.09, 126.18, 114.26, 86.18, 82.60, 79.49, 74.04, 73.94, 72.80, 62.55, 34.33, 26.06, 25.43, 14.24; *m/z* found [M$^+$] for $C_{23}H_{24}N_3O_9$ 486.1504 (calcd. 486.1513)

9: A mixture of compound **7** (0.062 g, 0.154 mmol), DCC (0.038 g, 0.184 mmol), and DMAP (0.001 g) were mixed in 7 mL of dry methylene chloride at 0° C. After 20 minutes, excess d-isosorbide (0.090 g, 0.616 mmol) was added and the solution stirred for 12 hours. The crude mixture was extracted with methylene chloride and washed twice with water and once with brine. The mixture was subjected to column chromatography (4:6 EtOAc:Hexane) yielding 60 mg (73% yield) of compound **9** as an orange solid. m.p. 130–131 °C; ^{1}H NMR (600 MHz, CD_2Cl_2) δ 12.17 (s, 1H), 8.10 – 7.95 (m, 2H), 7.82 – 7.68 (m, 2H), 5.44 (d, J = 3.5 Hz, 1H), 4.70 (t, J = 4.9 Hz, 1H), 4.61 (d, J = 4.5 Hz, 1H), 4.41 (q, J = 7.1 Hz, 2H), 4.32 (dq, J = 7.5, 5.8 Hz, 1H), 4.17 (d, J = 10.7 Hz, 1H), 4.10 (dd, J = 10.7, 3.7 Hz, 1H), 3.90 (dd, J = 9.4, 6.0 Hz, 1H), 3.59 (dd, J = 9.4, 5.9 Hz, 1H), 2.57 (d, J = 7.4 Hz, 1H), 1.37 (t, J = 7.1 Hz, 3H); ^{13}C NMR (151 MHz, CD_2Cl_2) δ 165.53, 163.26, 140.42, 134.15, 132.60, 131.20, 129.69, 129.52, 129.40, 128.79, 86.15, 82.57, 79.43, 74.03, 73.92, 73.85, 72.77, 62.57, 14.22. *m/z* found [M$^+$] for $C_{23}H_{20}N_2O_7F_5$ 531.1182 (calcd. 531.1191)

Scheme S3. Synthesis of asymmetric hydrazone dopants **1** and **2**.

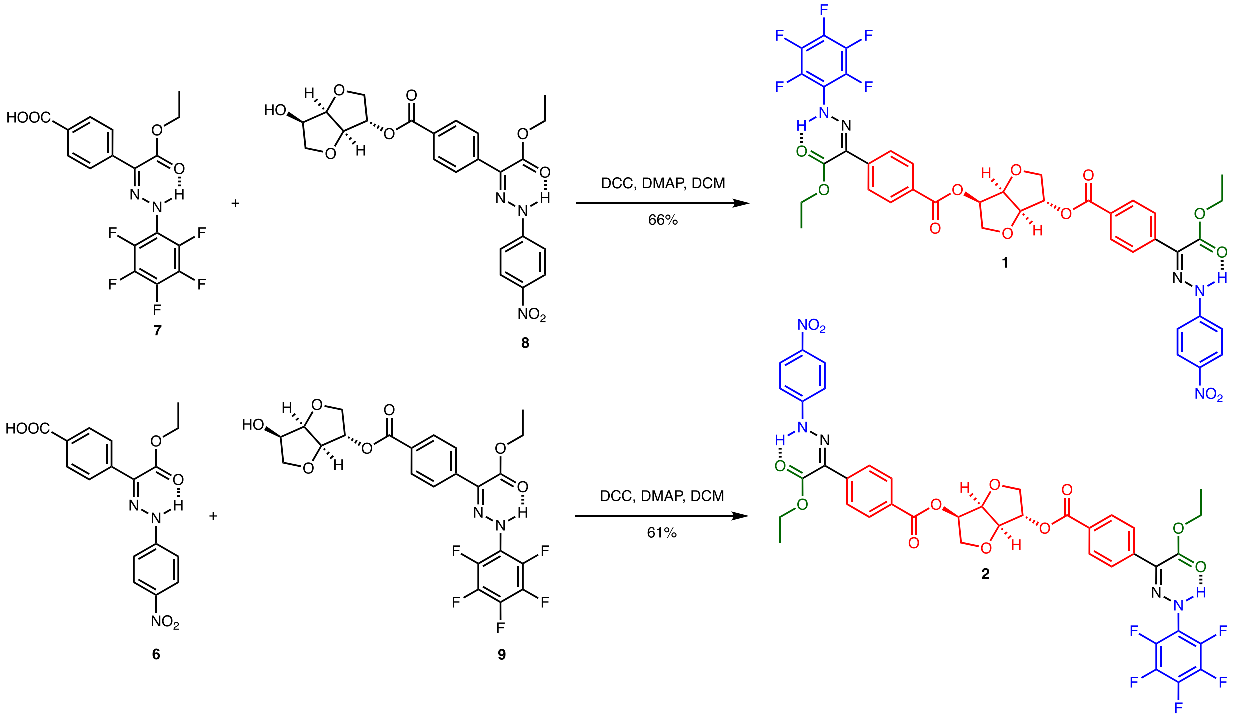

1: A mixture of compound **7** (0.022 g, 0.055 mmol), DCC (0.014 g, 0.066 mmol), and DMAP (0.001 g) were mixed in 5 mL of dry methylene chloride at 0° C. After 20 minutes, compound **8** (0.027 g, 0.055 mmol) was added and the solution stirred for 12 hours. The crude mixture was extracted with methylene chloride and washed twice with water and once with brine. The mixture was subjected to column chromatography (4:6 EtOAc:Hexane) yielding 32 mg of compound **1** (61 % yield) as a yellow solid. m.p. 112–113 °C; ^{1}H NMR (500 MHz, CD_2Cl_2) δ 12.63 (s, 1H), 12.17 (s, 1H), 8.22 (d, *J* = 9.0 Hz, 2H), 8.07 (t, *J* = 8.2 Hz, 4H), 7.79 (dd, *J* = 8.6, 2.5 Hz, 4H), 7.43 – 7.29 (m, 2H), 5.49 – 5.41 (m, 2H), 5.06 (t, *J* = 5.2 Hz, 1H), 4.69 (d, *J* = 4.9 Hz, 1H), 4.41 (dq, *J* = 9.4, 7.1 Hz, 4H), 4.15 – 4.04 (m, 4H), 1.41 – 1.34 (m, 6H); ^{19}F NMR (565 MHz, CD_3CN) δ -131.60 – -229.27 (m), -172.08; ^{13}C NMR (151 MHz, CD_2Cl_2) δ 165.77, 165.62, 163.43, 163.29, 148.53, 142.92, 140.72, 140.32, 132.66, 131.69, 129.70, 129.67, 129.58, 129.55, 129.07, 128.85, 126.16, 114.23, 86.66, 81.64, 79.10, 75.02, 73.75, 71.35, 62.57, 62.53, 14.38, 14.22; *m/z* found [M^-] for $C_{40}H_{31}N_5O_{12}F_5$ 868.1903 (calcd. 868.1889)

2: A mixture of compound **6** (0.032 g, 0.090 mmol), DCC (0.019 g, 0.092 mmol), and DMAP (0.001 g) were mixed in 5 mL of dry methylene chloride at 0° C. After 20 minutes, compound **9**

(0.040 g, 0.075 mmol) was added and the solution stirred for 12 hours. The crude mixture was extracted with methylene chloride and washed twice with water and once with brine. The mixture was subjected to column chromatography (4:6 EtOAc:Hexane) yielding 40 mg (61 % yield) of compound **2** as a yellow-orange solid. m.p. 109–110 °C; ^{1}H NMR (500 MHz, CD_2Cl_2) δ 12.68 (s, 1H), 12.20 (s, 1H), 8.27 (d, J = 9.0 Hz, 2H), 8.14 (d, J = 8.3 Hz, 2H), 8.07 (d, J = 8.3 Hz, 2H), 7.85 (d, J = 8.2 Hz, 2H), 7.80 (d, J = 8.3 Hz, 2H), 7.45 – 7.36 (m, 2H), 5.52 – 5.47 (m, 2H), 5.10 (t, J = 5.2 Hz, 1H), 4.73 (d, J = 4.9 Hz, 1H), 4.49 – 4.41 (m, 4H), 4.20 – 4.09 (m, 4H), 1.44 – 1.38 (m, 6H). ^{19}F NMR (565 MHz, CD_2Cl_2) *d* -155.98 (d, J = 21.0 Hz), -163.59 (td, J = 21.5, 5.6 Hz), -165.05 (t, J = 21.6 Hz); ^{13}C NMR (151 MHz, CD_2Cl_2) δ 165.76, 165.63, 163.46, 163.40, 148.56, 142.93, 140.65, 140.41, 132.63, 131.77, 129.70, 129.68, 129.62, 129.55, 129.14, 128.79, 126.17, 114.24, 86.67, 81.66, 79.08, 75.06, 73.78, 71.36, 62.57, 62.54, 14.24, 14.21. *m/z* found [M^-] for $C_{40}H_{31}N_5O_{12}F_5$ 868.1901 (calcd. 868.1889)

Scheme S4. Synthesis of symmetric hydrazone dopants **3** and **4**.

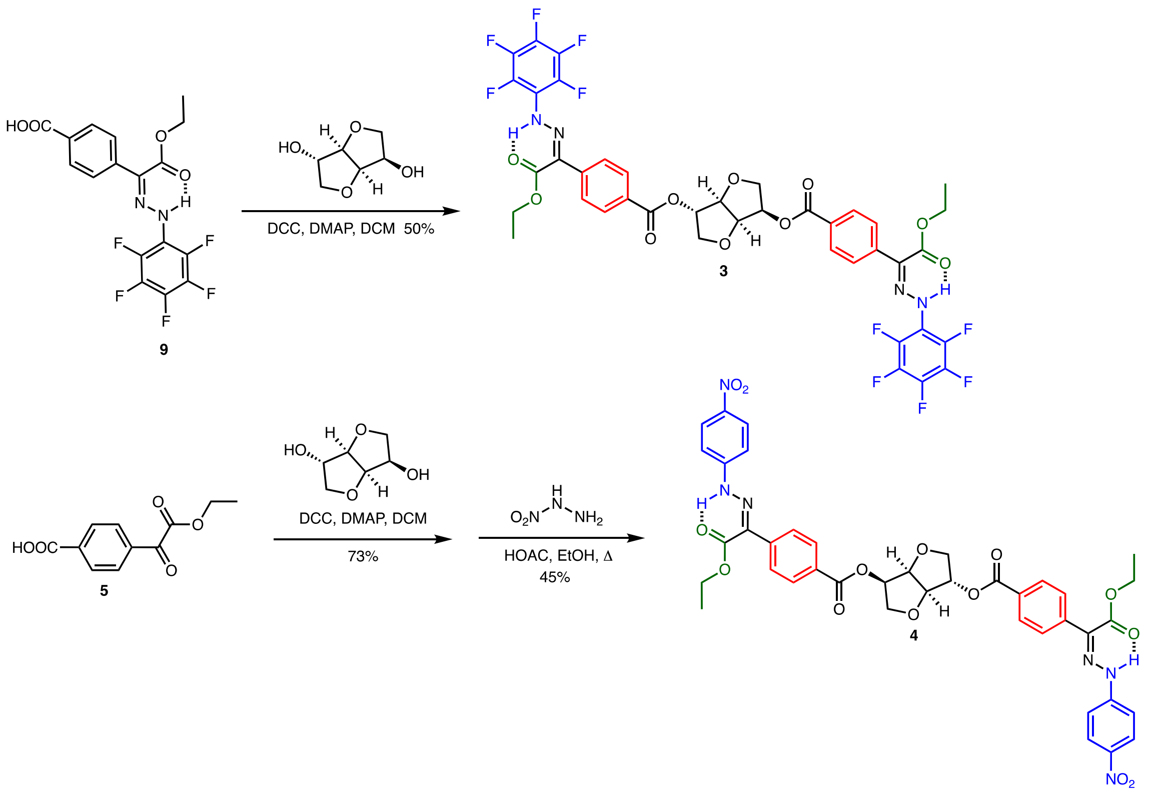

3: This compound was synthesized by following the procedure used for compound **1**. Compound **3** was obtained in 50% yield (0.05 g) as a white solid. m.p. 173–174 °C; ^{1}H NMR (600 MHz, CD_2Cl_2) δ 12.16 (dt, *J* = 4.9, 2.1 Hz, 2H), 8.09 – 8.05 (m, 2H), 8.05 – 8.01 (m, 2H), 7.80 – 7.74 (m, 4H), 5.47 – 5.45 (m, 1H), 5.45 – 5.42 (m, 1H), 5.05 (t, *J* = 5.2 Hz, 1H), 4.68 (d, *J* = 4.9 Hz, 1H), 4.46 – 4.38 (m, 4H), 4.12 (dd, *J* = 10.7, 3.4 Hz, 1H), 4.09 (s, 1H), 4.06 (t, *J* = 5.2 Hz, 2H), 1.38 (q, *J* = 7.0 Hz, 6H). ^{19}F NMR (565 MHz, CD_3CN) δ -156.95 (d, *J* = 21.7 Hz), -165.24 (t, *J* = 20.1 Hz), -166.66 (t, *J* = 21.0 Hz); ^{13}C NMR (151 MHz, CD_2Cl_2) δ 165.78, 165.64, 163.31, 163.28, 140.41, 140.33, 137.99, 132.72, 132.66, 131.22, 129.71, 129.69, 129.63, 129.58, 129.40, 128.86, 128.80, 86.69, 81.66, 79.10, 75.04, 73.77, 71.35, 62.58, 54.20, 54.02, 53.84, 53.66, 53.48, 14.24, 14.22. *m/z* found [M^-] for $C_{40}H_{28}N_4O_{10}F_{10}$ 913.1578 (calcd. 913.1568).

4: This compound was synthesized by following a reported procedure.[S1] Compound **4** was obtained in 45% yield (0.023 g) and its identity confirmed by comparing its ^{1}H NMR spectrum with the reported one; ^{1}H NMR (600 MHz, CD_2Cl_2) δ 12.63 (d, *J* = 4.4 Hz, 2H), 8.23 (dd, *J* = 9.2, 2.9 Hz, 4H), 8.10 (d, *J* = 8.2 Hz, 2H), 8.06 (d, *J* = 8.2 Hz, 2H), 7.81 (dd, *J* = 12.8, 8.2 Hz, 4H), 7.38 (dd, *J* = 9.0, 4.8 Hz, 4H), 5.50 – 5.44 (m, 2H), 5.07 (t, *J* = 5.3 Hz, 1H), 4.70 (d, *J* = 5.0 Hz, 1H), 4.41 (p, *J* = 7.0 Hz, 4H), 4.14 – 4.05 (m, 4H), 1.37 (q, *J* = 7.1 Hz, 6H).

3. NMR Characterization

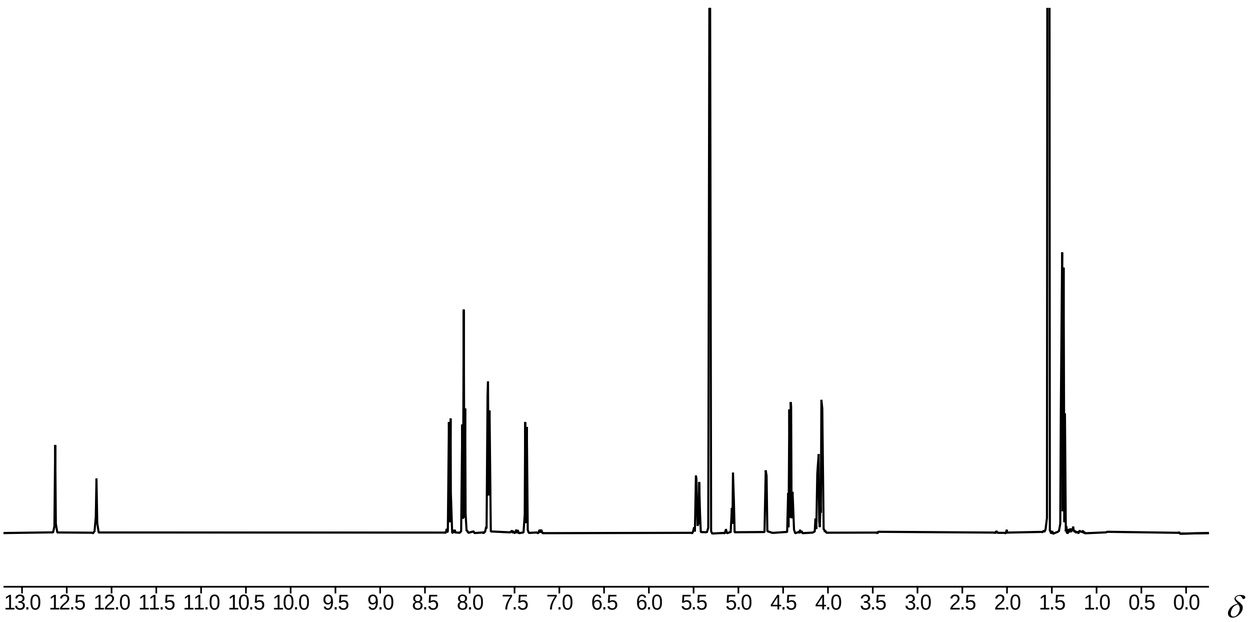

Figure S1: ^{1}H NMR spectrum of compound **1** in CD_2Cl_2 at 298 K.

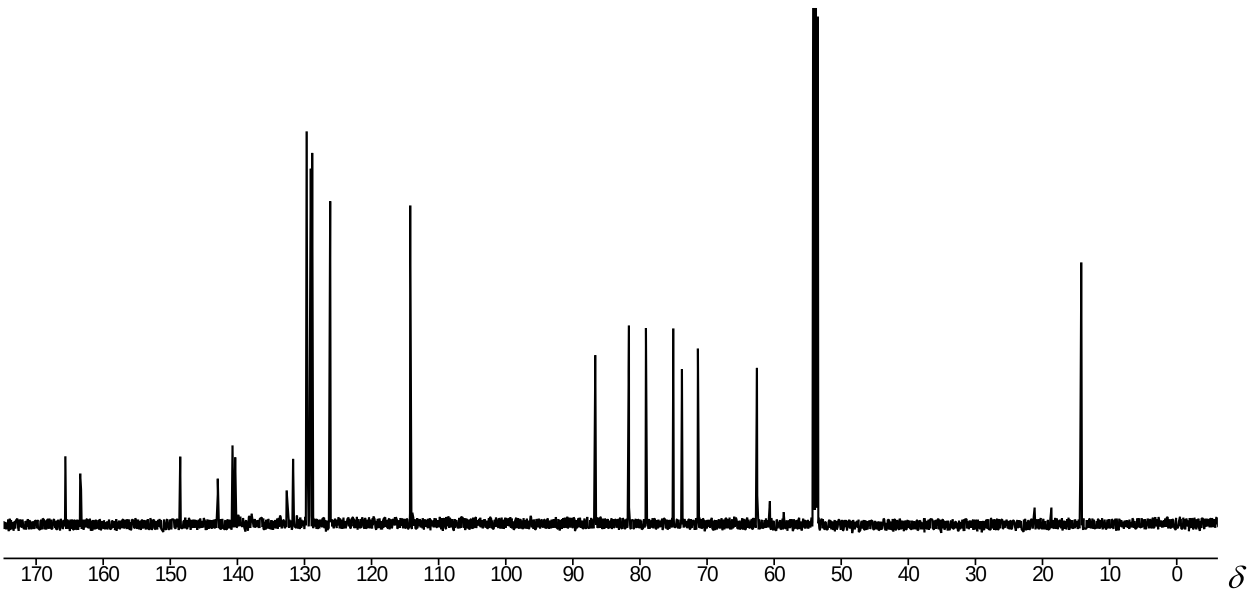

Figure S2: ^{13}C NMR spectrum of compound **1** in CD_2Cl_2 at 298 K.

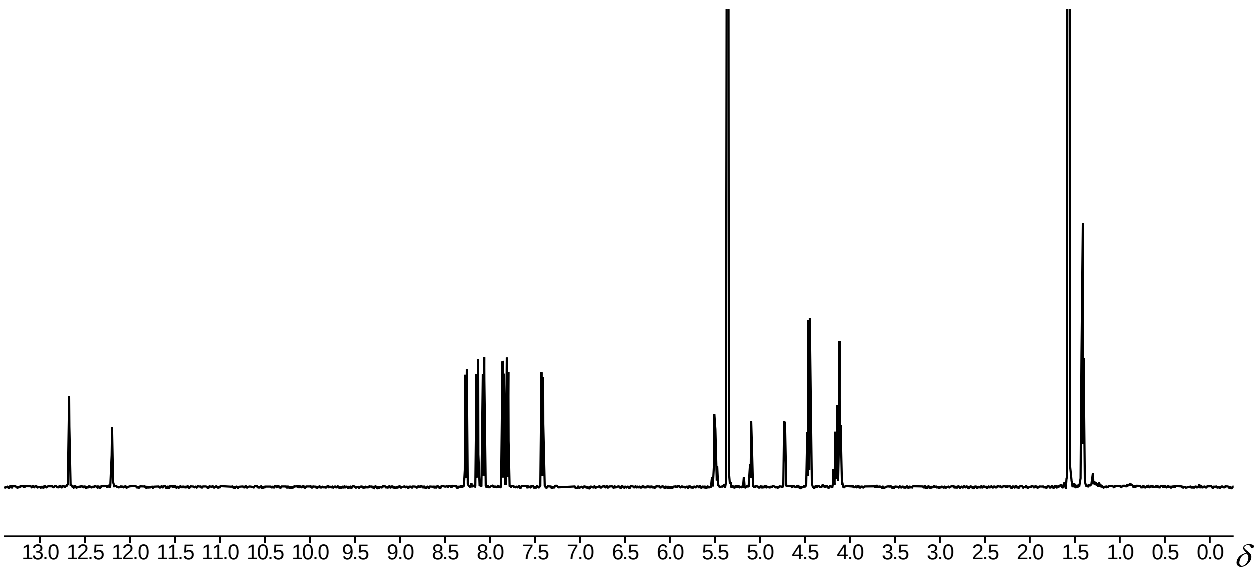

Figure S3: ^{1}H NMR spectrum of compound **2** in CD_2Cl_2 at 298 K.

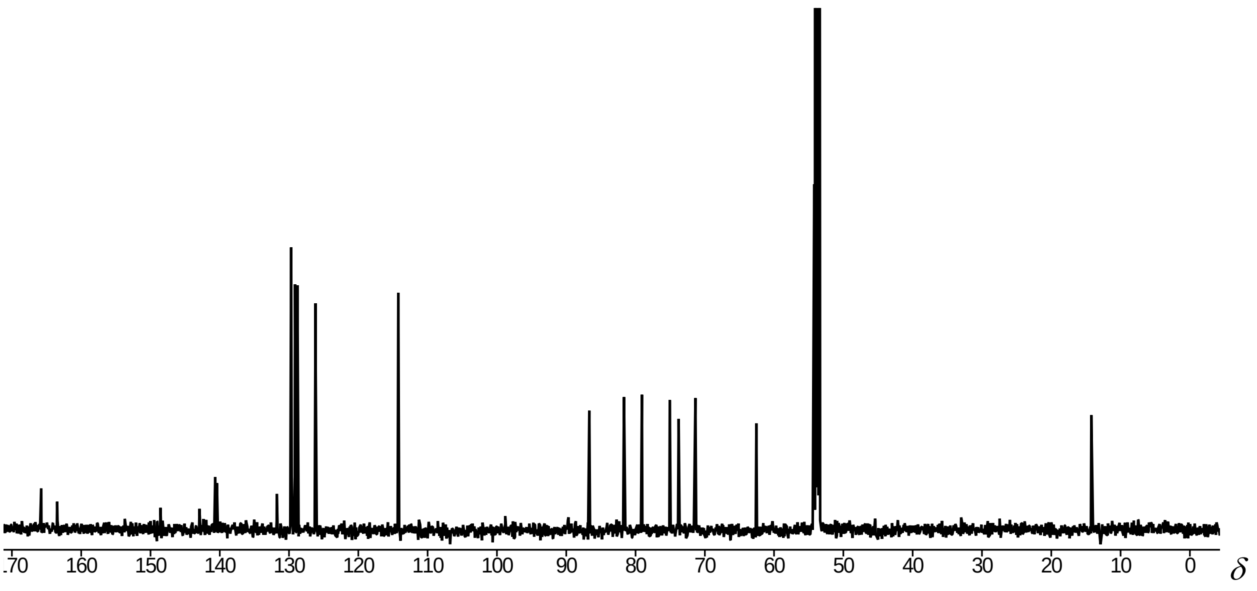

Figure S4: ^{13}C NMR spectrum of compound **2** in CD_2Cl_2 at 298 K.

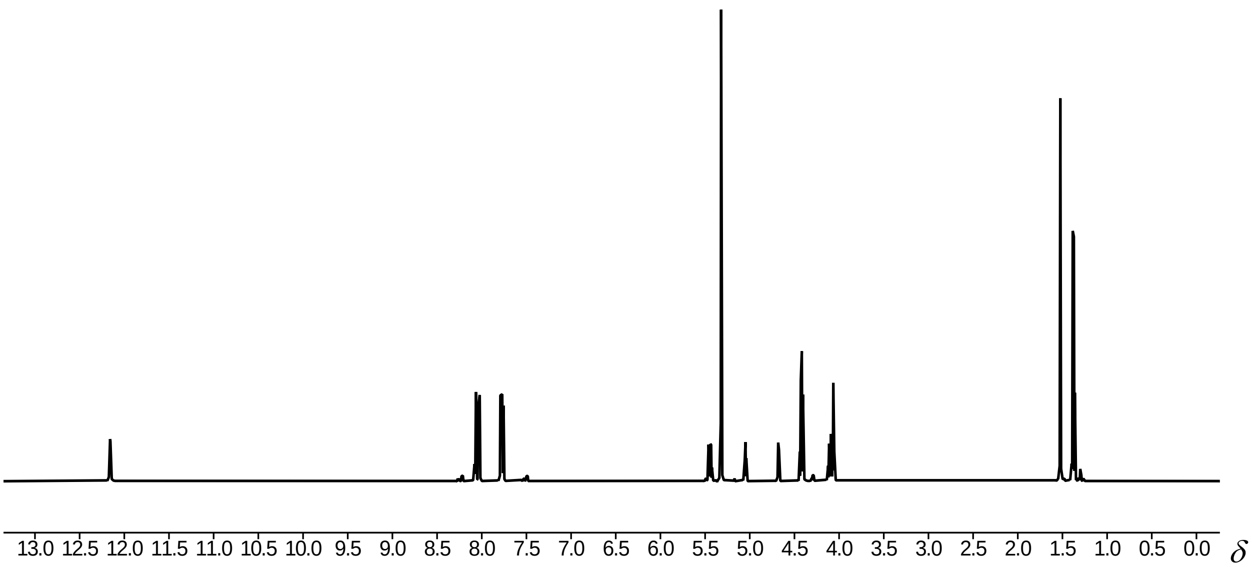

Figure S5: ^{1}H NMR spectrum of compound **3** in CD_2Cl_2 at 298 K.

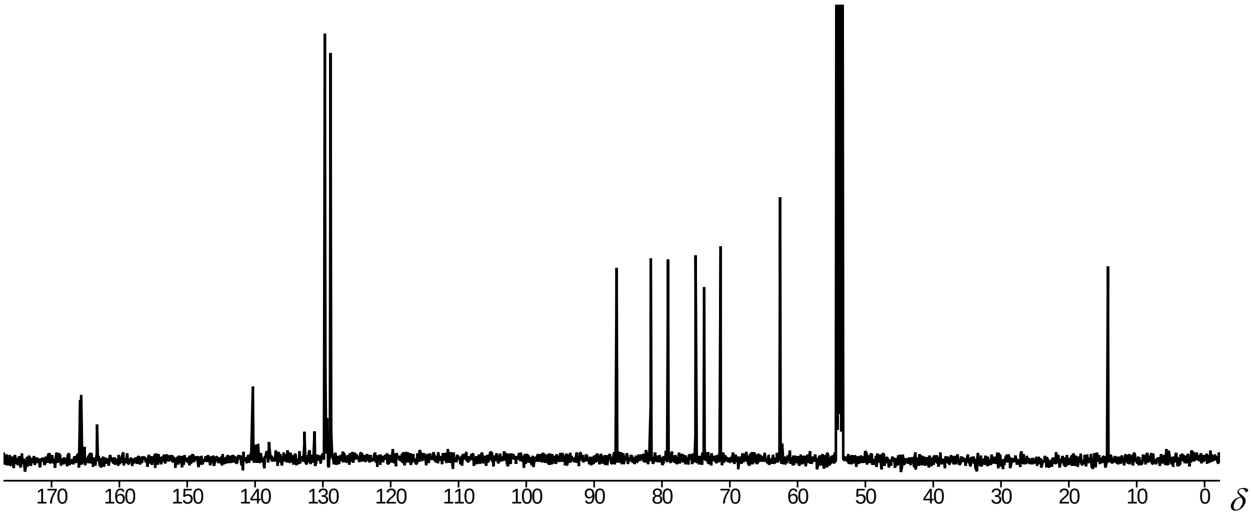

Figure S6: ^{13}C NMR spectrum of compound **3** in CD_2Cl_2 at 298 K.

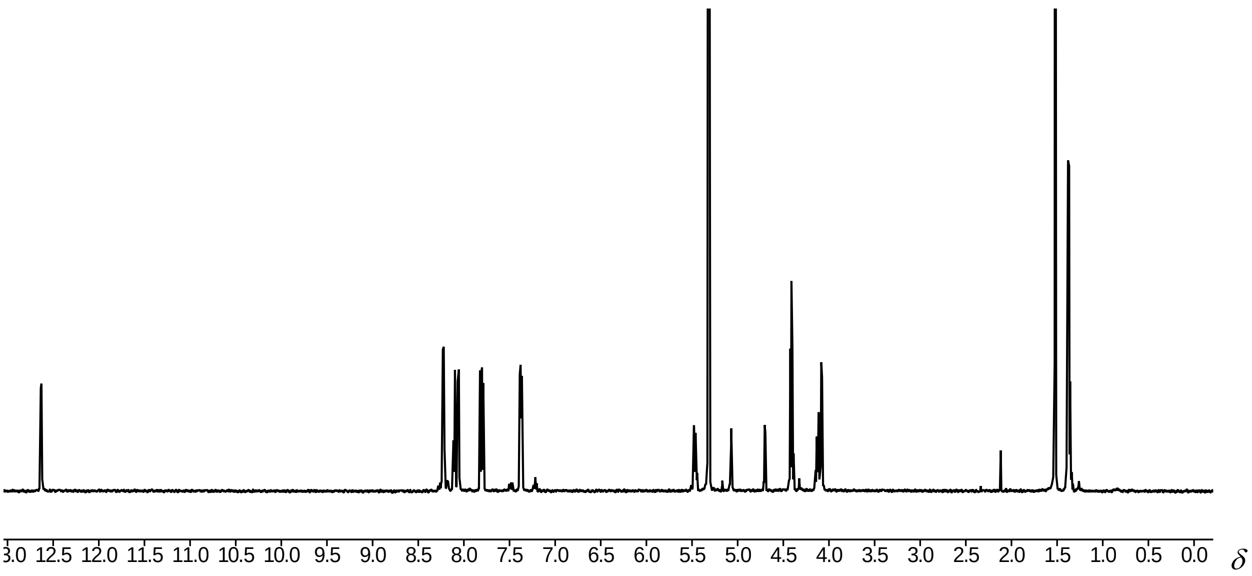

Figure S7: ^{1}H NMR spectrum of compound **4** in CD_2Cl_2 at 298 K.

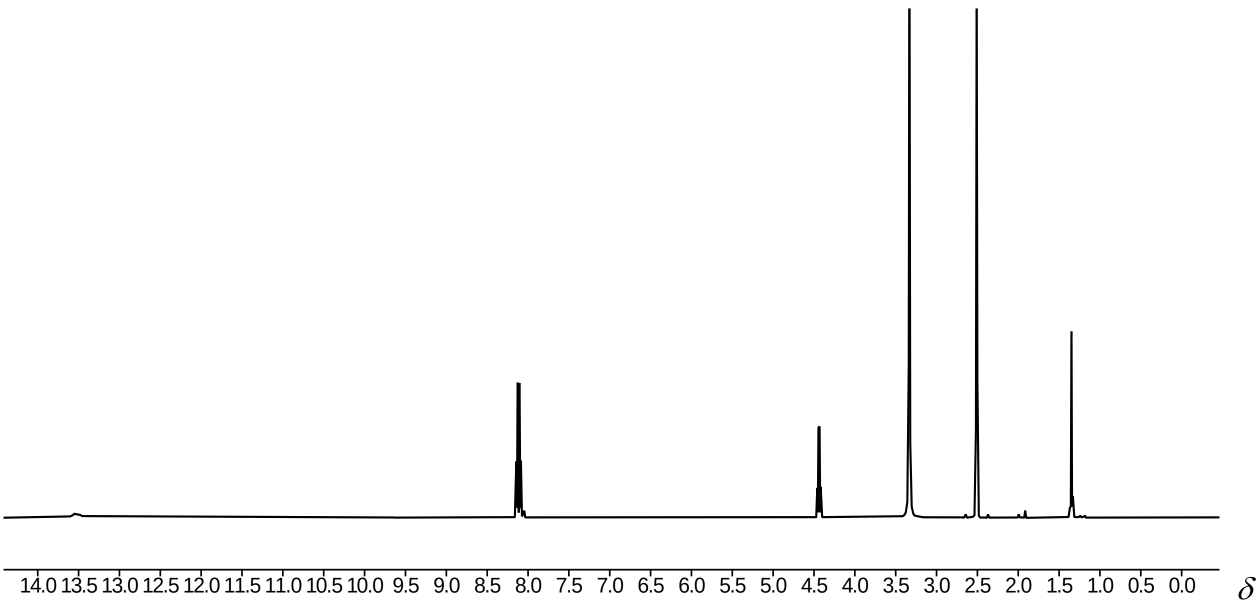

Figure S8: ^{1}H NMR spectrum of compound **5** in DMSO at 298 K.

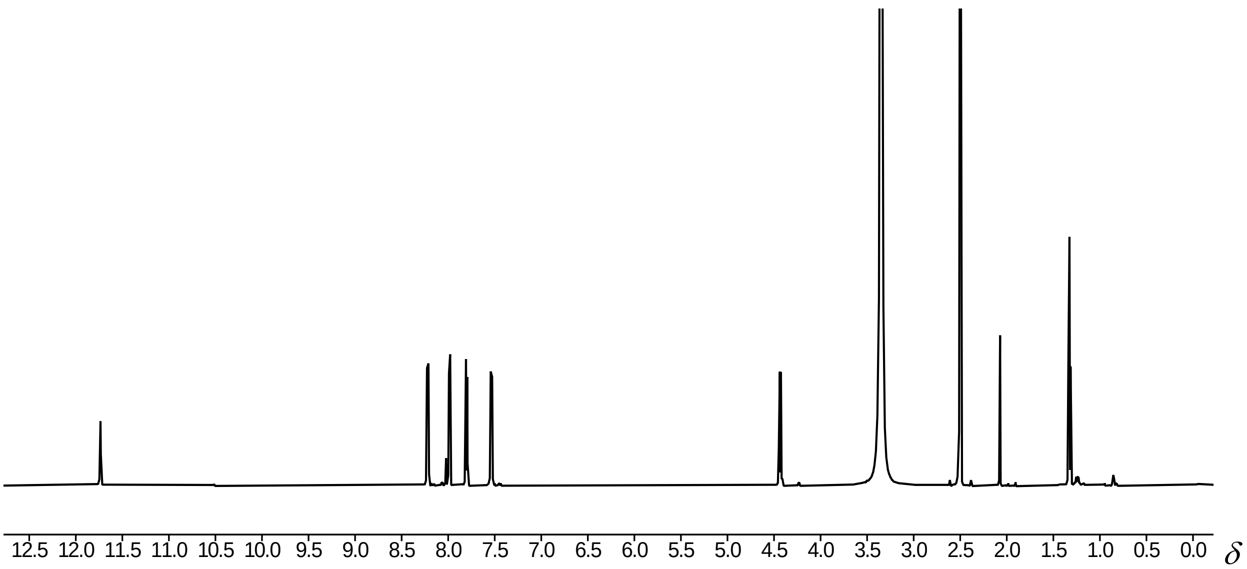

Figure S9: ^{1}H NMR spectrum of compound **6** in DMSO at 298 K.

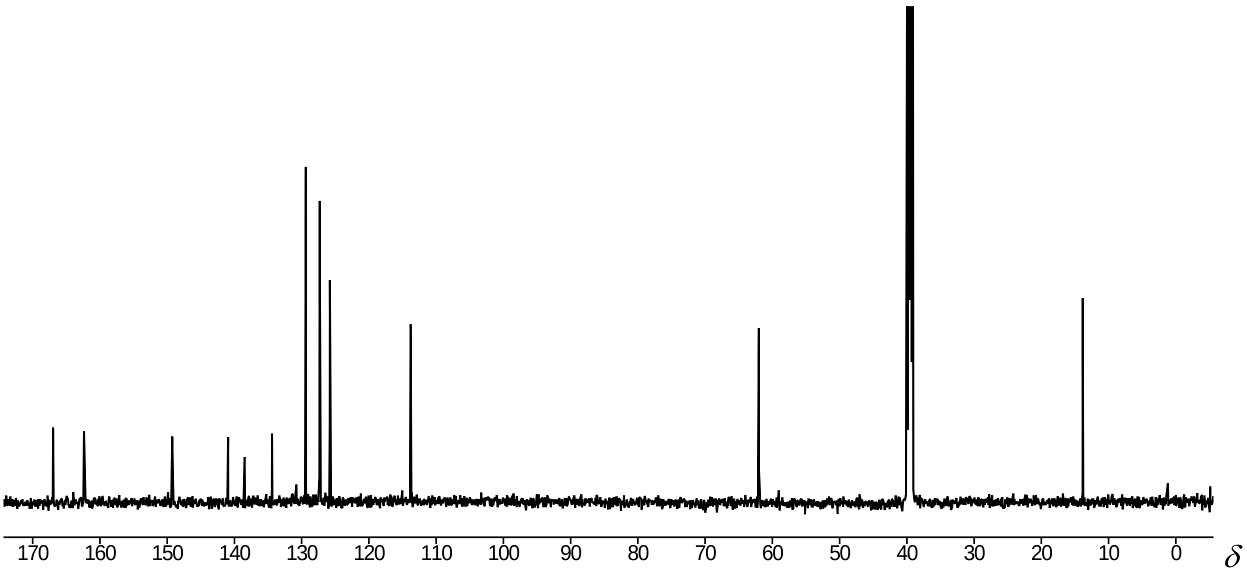

Figure S10: ^{13}C NMR spectrum of compound **6** in DMSO at 298 K.

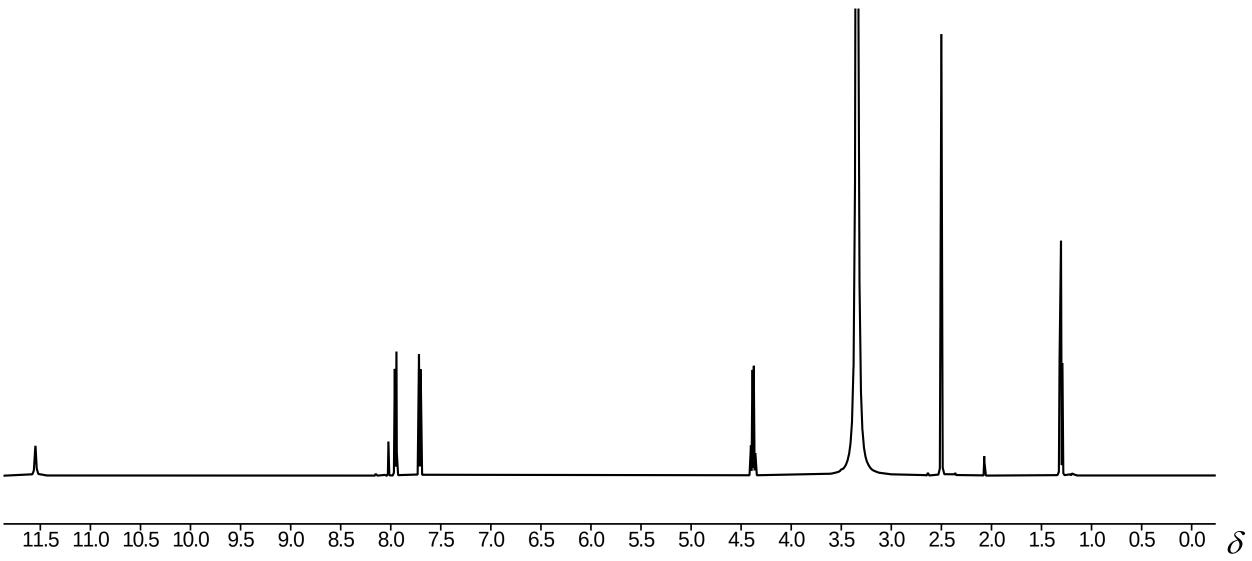

Figure S11: ^{1}H NMR spectrum of compound **7** in DMSO at 298 K.

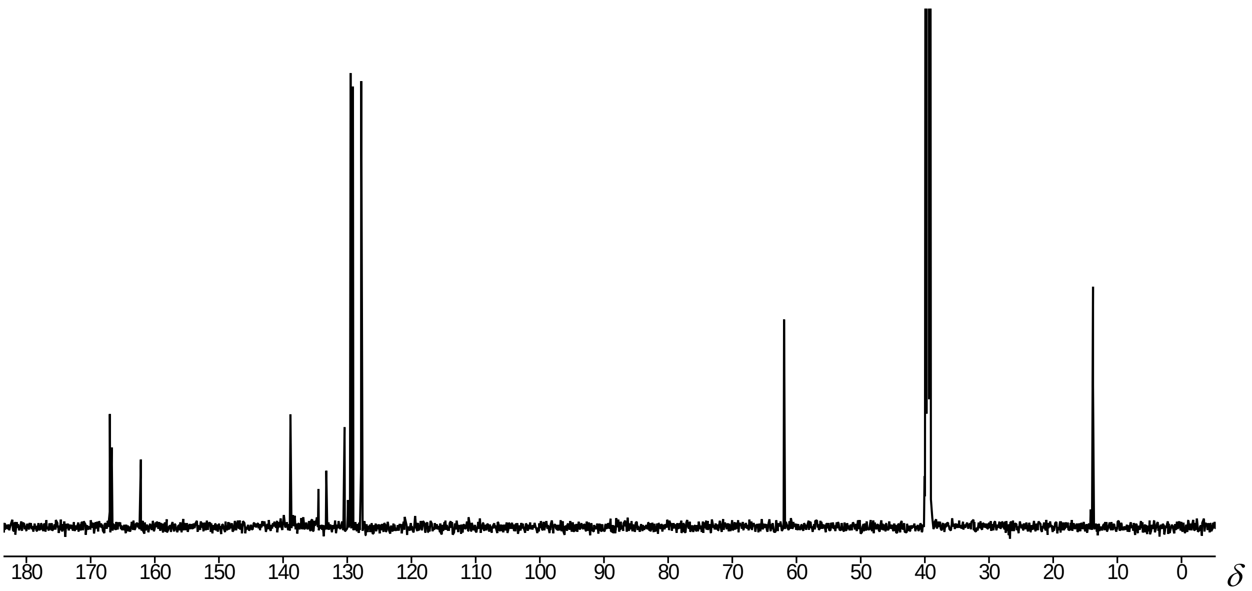

Figure S12: ^{13}C NMR spectrum of compound **7** in DMSO at 298 K.

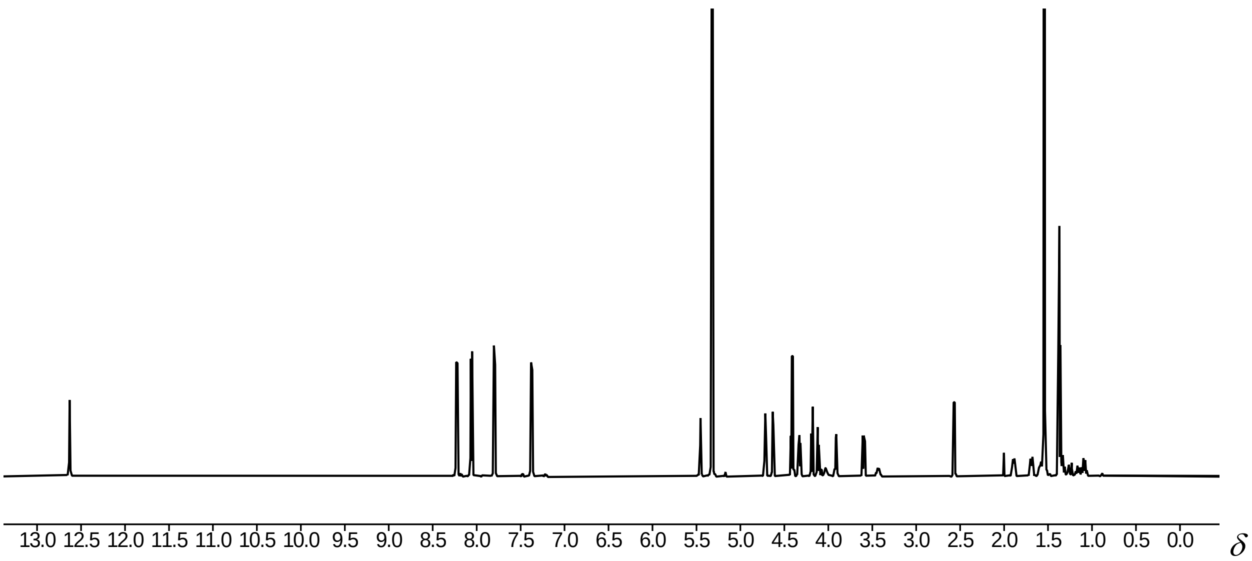

Figure S13: ^{1}H NMR spectrum of compound **8** in CD_2Cl_2 at 298 K.

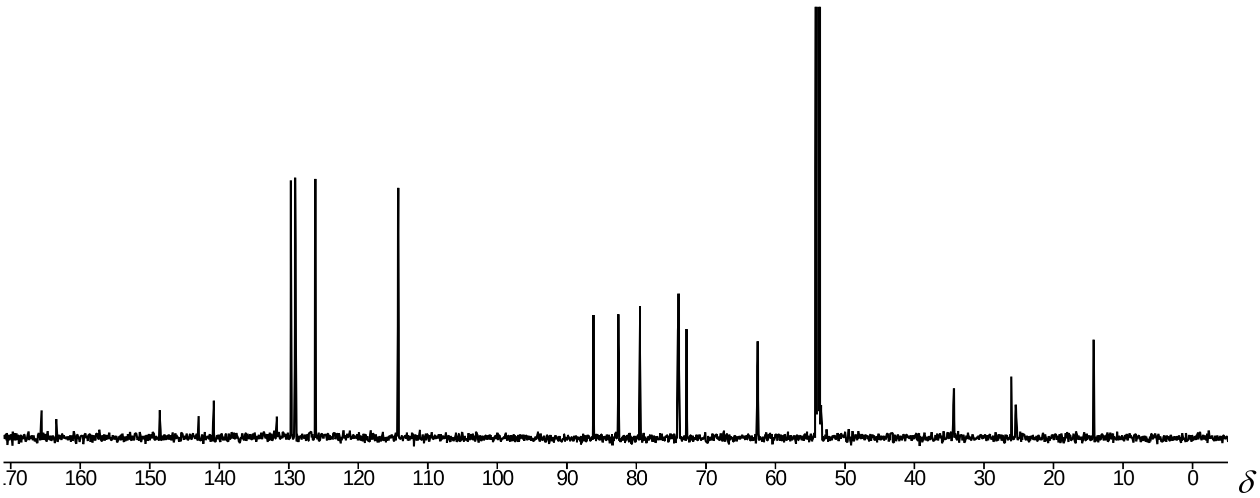

Figure S14: ^{13}C NMR spectrum of compound **8** in CD_2Cl_2 at 298 K.

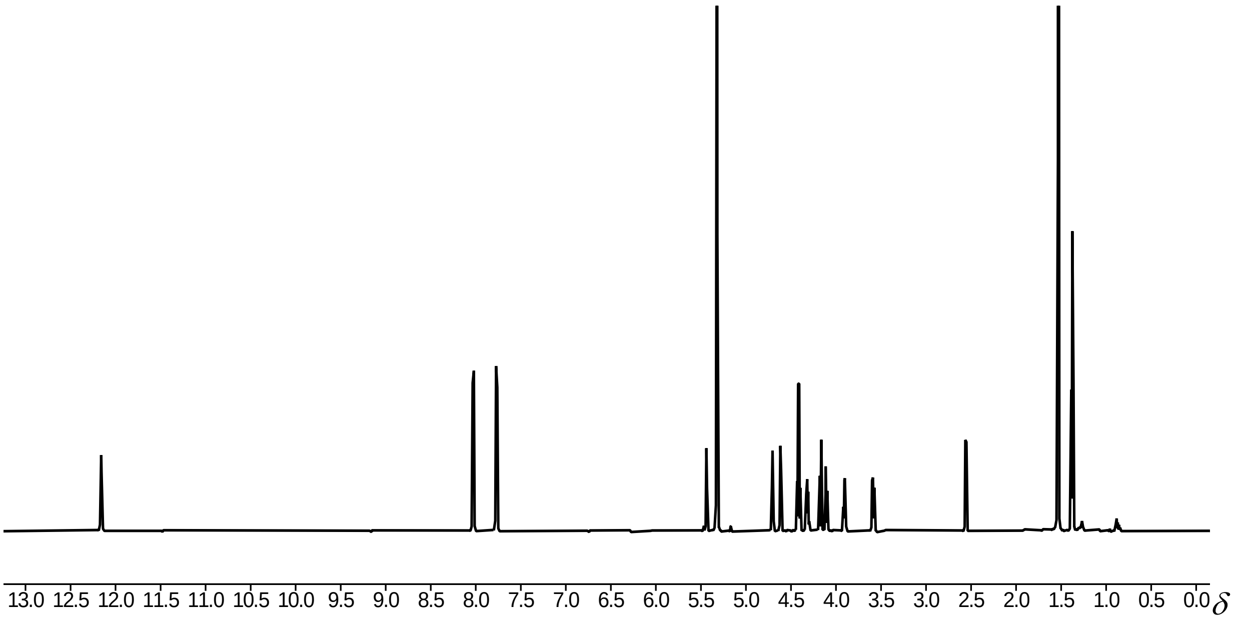

Figure S15: ^{1}H NMR spectrum of compound **9** in CD_2Cl_2 at 298 K.

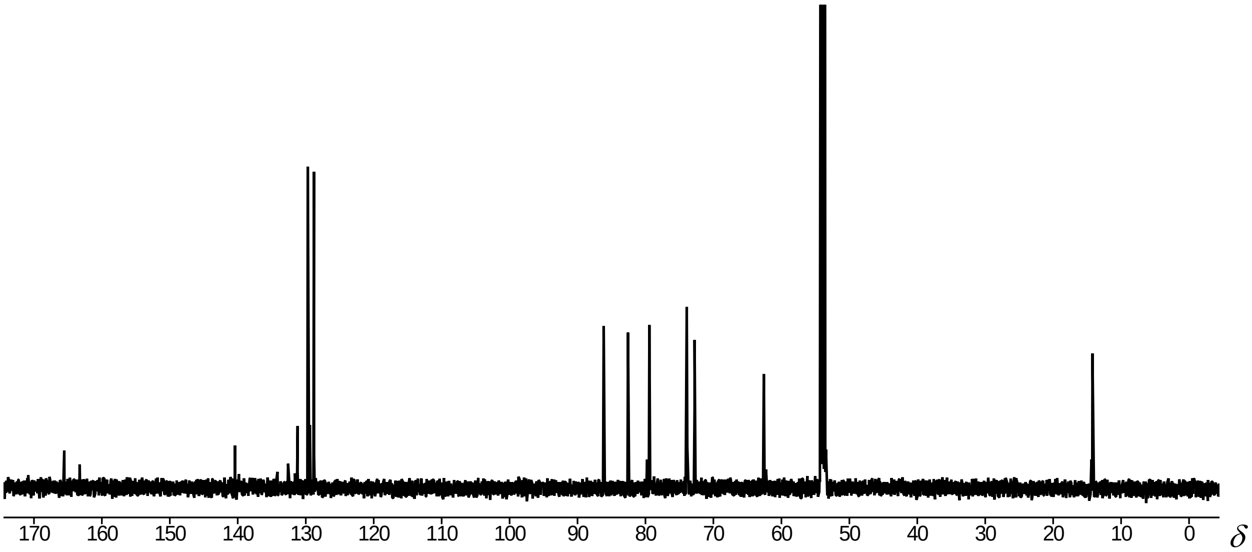

Figure S16: ^{13}C NMR spectrum of compound **9** in CD_2Cl_2 at 298 K.

4. Photoisomerization Studies

UV-Vis, Circular dichroism (CD) and ^{1}H NMR spectroscopies were employed to study the photoisomerization of the hydrazone switches. Spectrophotometric grade solvents were used for the absorption studies. Hydrazone switch solutions in MeCN (3.0 mL, 1.0×10^{-5} M) were prepared and transferred into a 1.0 cm quartz cuvette for immediate UV/Vis absorption measurements. The solutions were then irradiated, and their UV spectra recorded. Isomerization cycles were measured by alternating the irradiation wavelength between the appropriate wavelengths and monitoring the change in UV/Vis absorption. The photostationary states (PSS) were determined upon continuous irradiation of the sample until no further isomerization was observed using ^{1}H NMR spectroscopy. Hydrazone switch solutions in MeCN (100 μL, 2.5×10^{-4} M) were prepared and transferred into a 1.0 mm quartz cuvette for immediate CD measurements. The solutions were then irradiated, and their CD and UV spectra recorded. Photophysical data for hydrazone **4** was obtained following previous procedures.[S1]

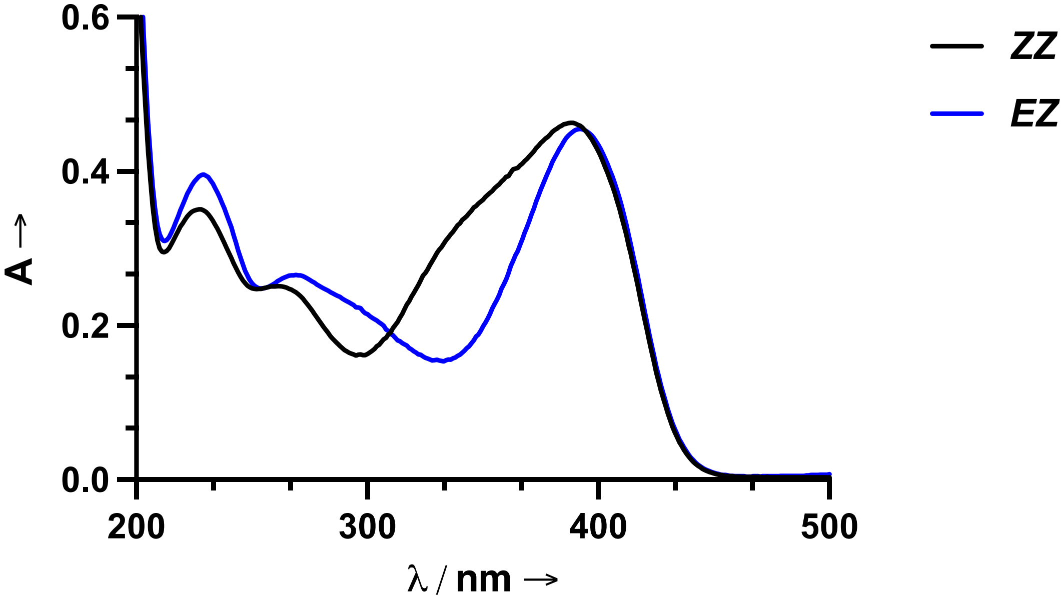

Figure S17: UV-Vis absorption spectra of **1**-*ZZ* and **1**-*EZ* in MeCN (1.0 x 10^{-5} M): The UV spectrum of **1**-*EZ* was obtained upon 340 nm light irradiation of the pristine sample.

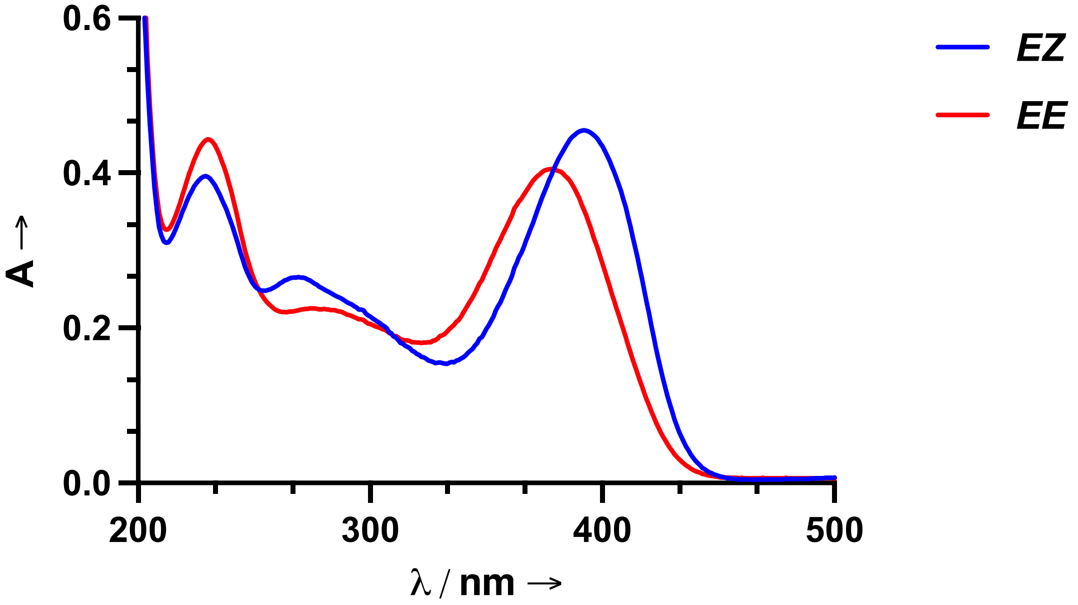

Figure S18: UV-Vis absorption spectra of **1**-*EZ* and **1**-*EE* in MeCN (1.0 x 10^{-5} M): The UV spectrum of **1**-*EE* was obtained upon 442 nm light irradiation of **1**-*EZ*.

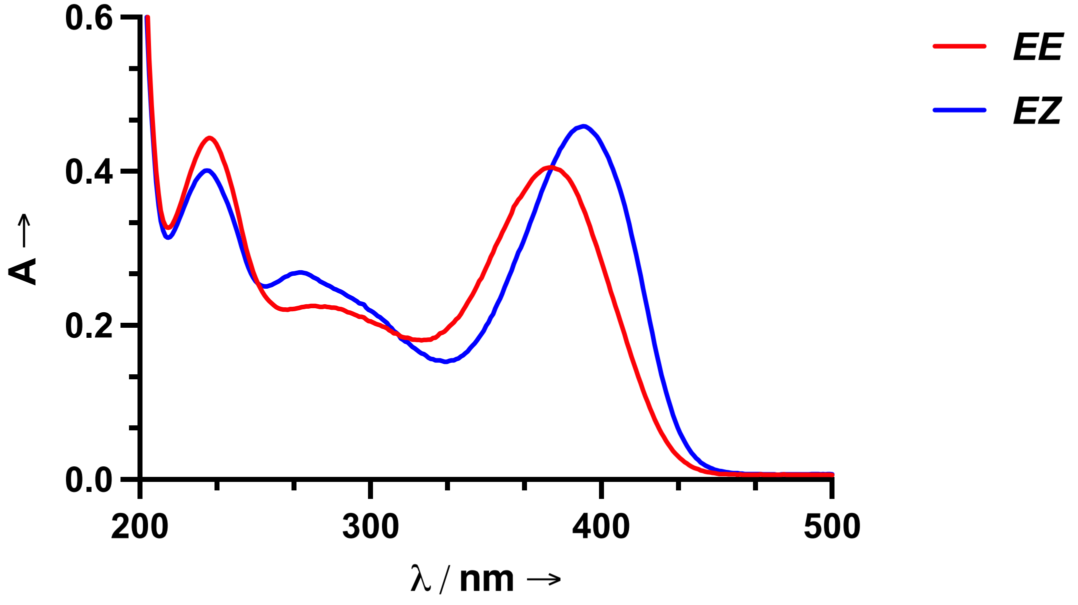

Figure S19: UV-Vis absorption spectra of **1**-*EE* and **1**-*EZ* in MeCN (1.0 x 10^{-5} M): The UV spectrum of **1**-*EZ* was obtained upon 340 nm light irradiation of **1**-*EE*.

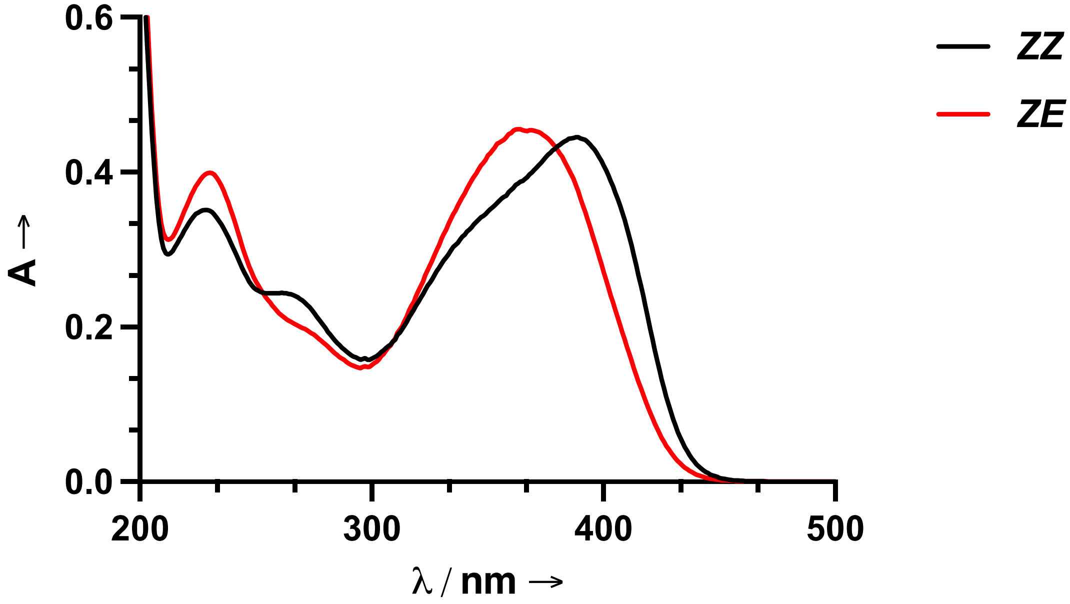

Figure S20: UV-Vis absorption spectra of **1**-*ZZ* and **1**-*ZE* in MeCN (1.0 x 10^{-5} M): The UV spectrum of **1**-*ZE* was obtained upon 442 nm light irradiation of the pristine sample.

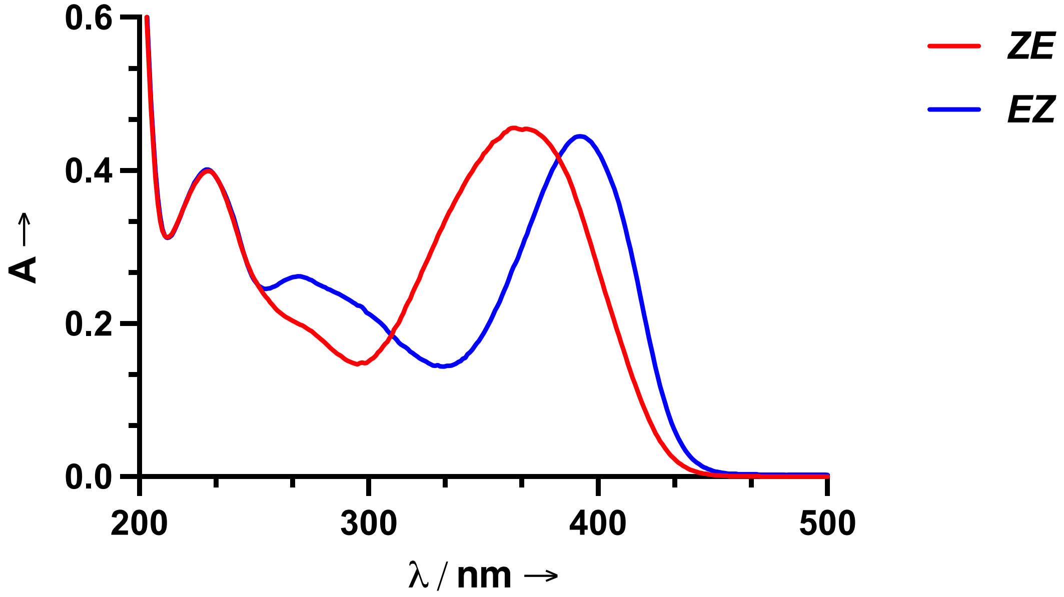

Figure S21: UV-Vis absorption spectra of **1**-*ZE* and **1**-*EZ* in MeCN (1.0 x 10^{-5} M): The UV spectrum of **1**-*EZ* was obtained upon 340 nm light irradiation of **1**-*ZE*.

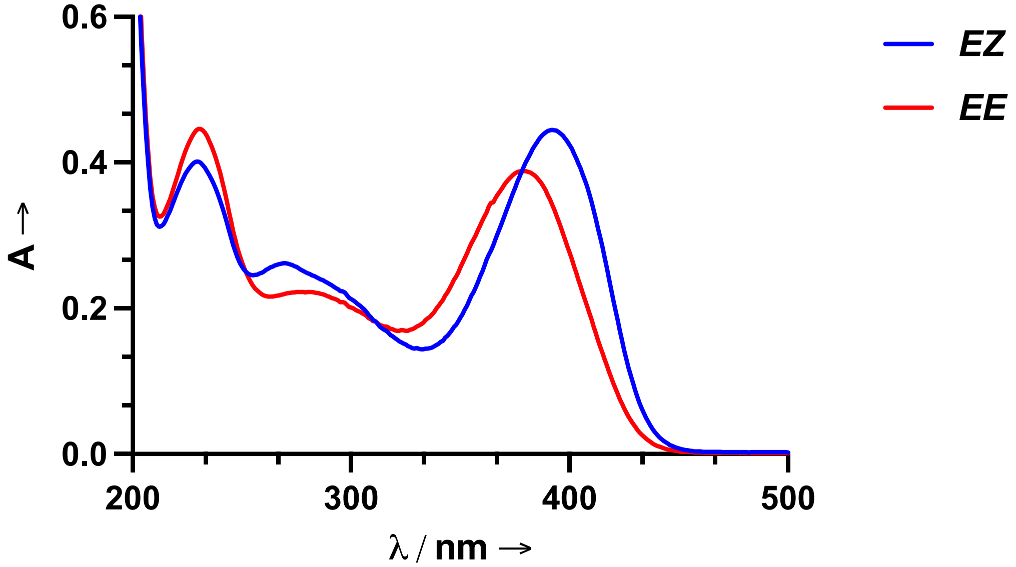

Figure S22: UV-Vis absorption spectra of **1**-*EZ* and **1**-*EE* in MeCN (1.0 x 10^{-5} M): The UV spectrum of **1**-*EE* was obtained upon 442 nm light irradiation of **1**-*EZ*.

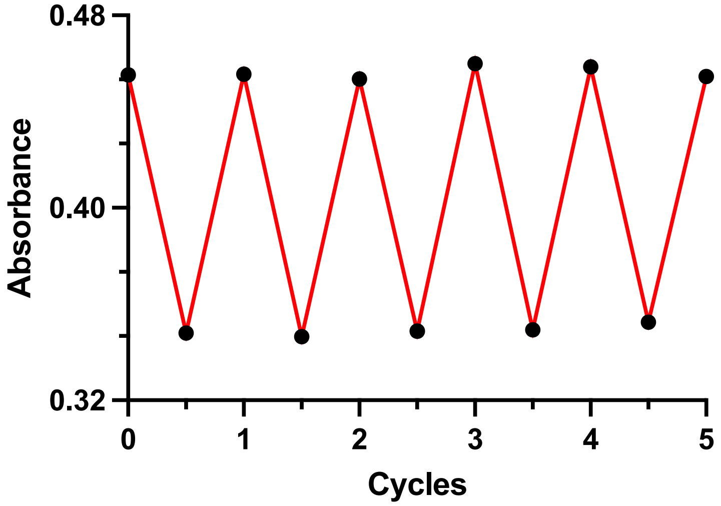

Figure S23: Photoisomerization cycles of hydrazone **1** in MeCN (1.0 × 10^{-5} M) at 294 K. The absorbance change of the *EE* and *EZ* state at 392 nm was monitored while alternating the irradiation wavelength between 340 and 442 nm.

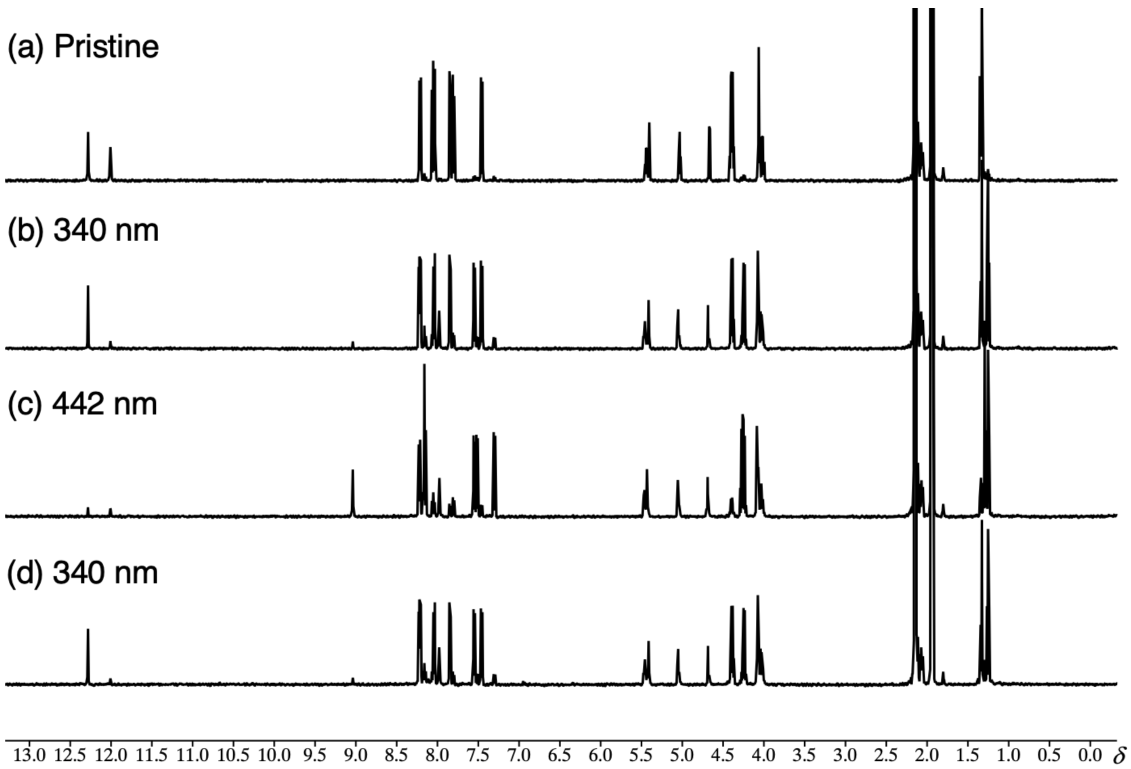

Figure S24: ^{1}H NMR spectra of (a) the pristine sample and the (b) 340, (c) 442, and (d) 340 nm photostationary states of **1** after sequential irradiation in CD_3CN at 294K.

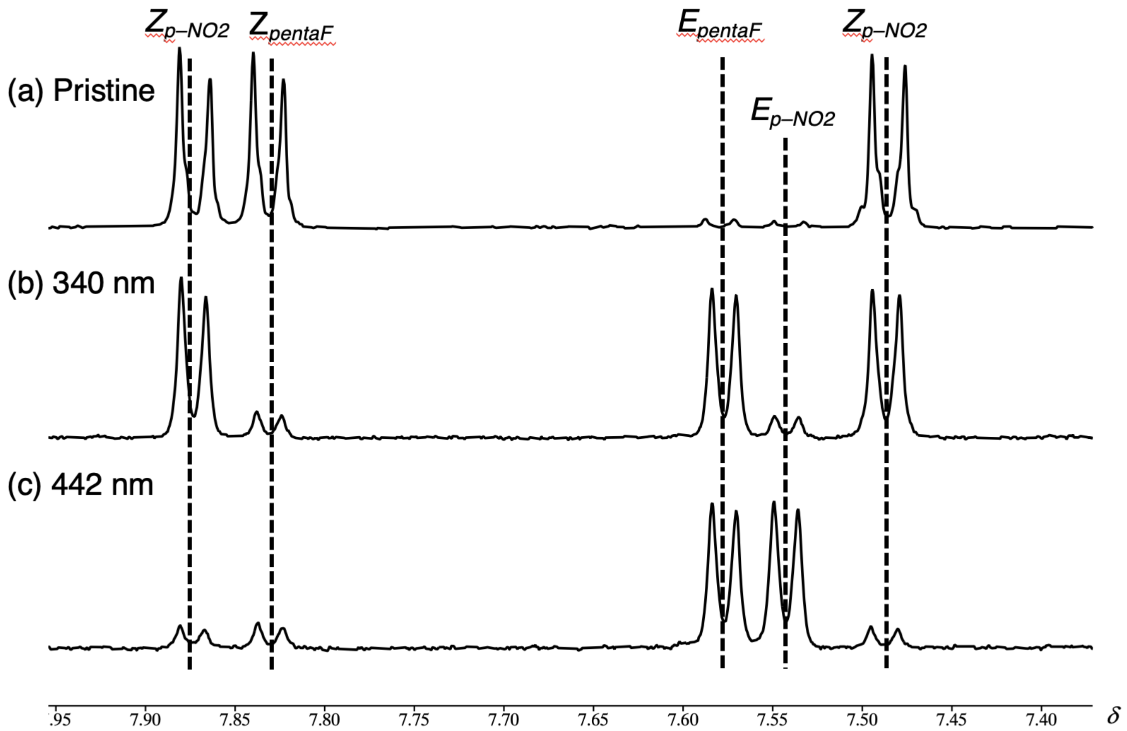

Figure S25: Zoom in on the ^{1}H NMR spectra of (a) the pristine sample and the (b) 340, (c) 442 nm photostationary states of **1** after sequential irradiation in CD_3CN at 294K.

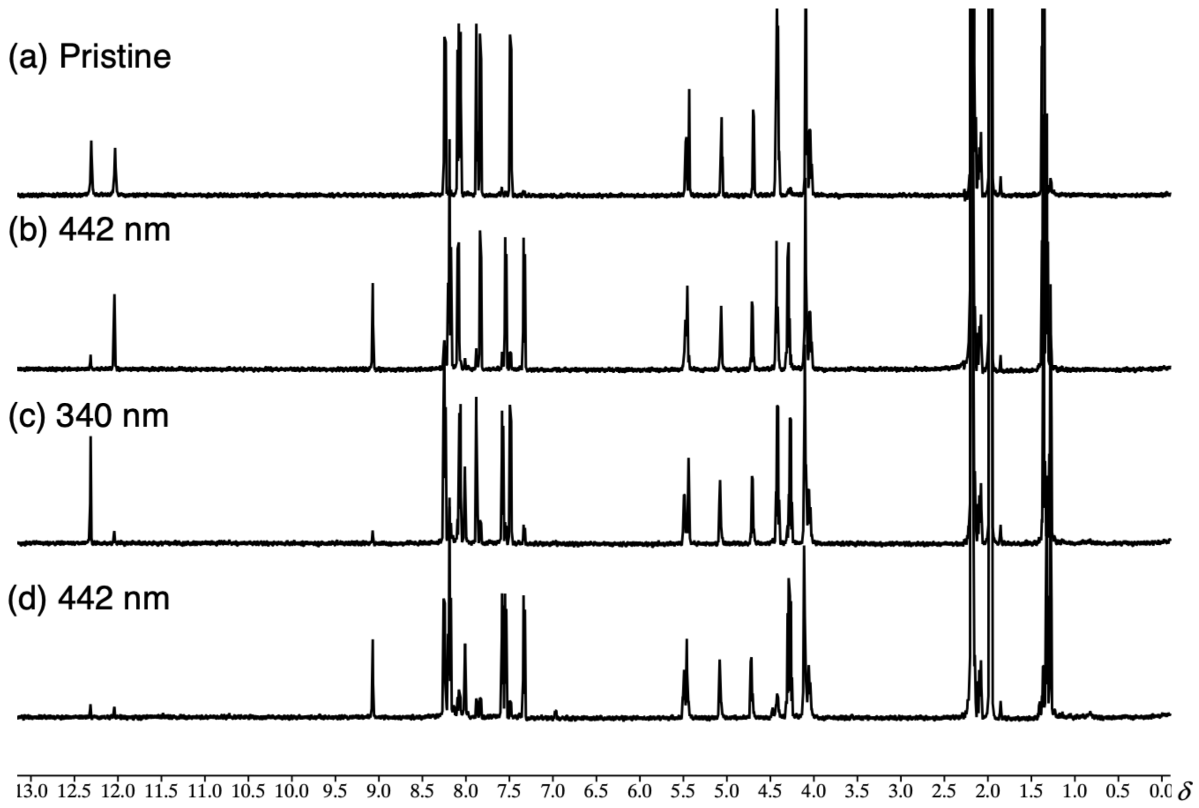

Figure S26: ^{1}H NMR spectra of (a) the pristine sample and the (b) 442, (c) 340, and (d) 442 nm photostationary states of **1** after sequential irradiation in CD_3CN at 294K.

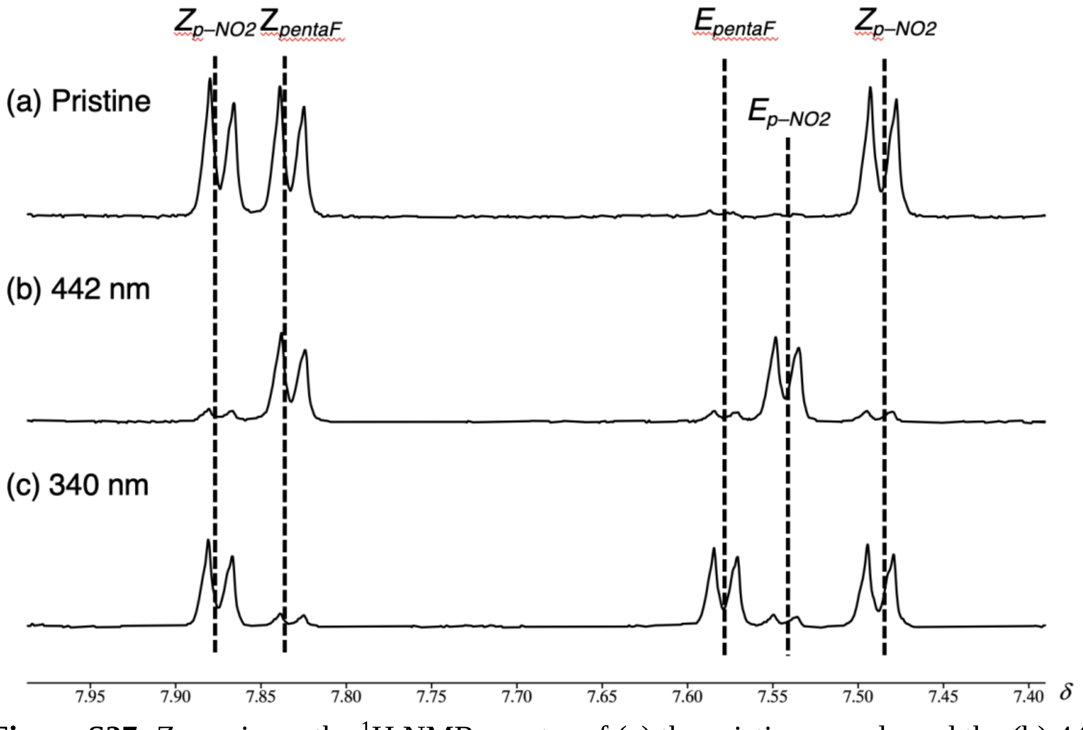

Figure S27: Zoom in on the ^{1}H NMR spectra of (a) the pristine sample and the (b) 442, (c) 340 nm photostationary states of **1** after sequential irradiation in CD_3CN at 294K.

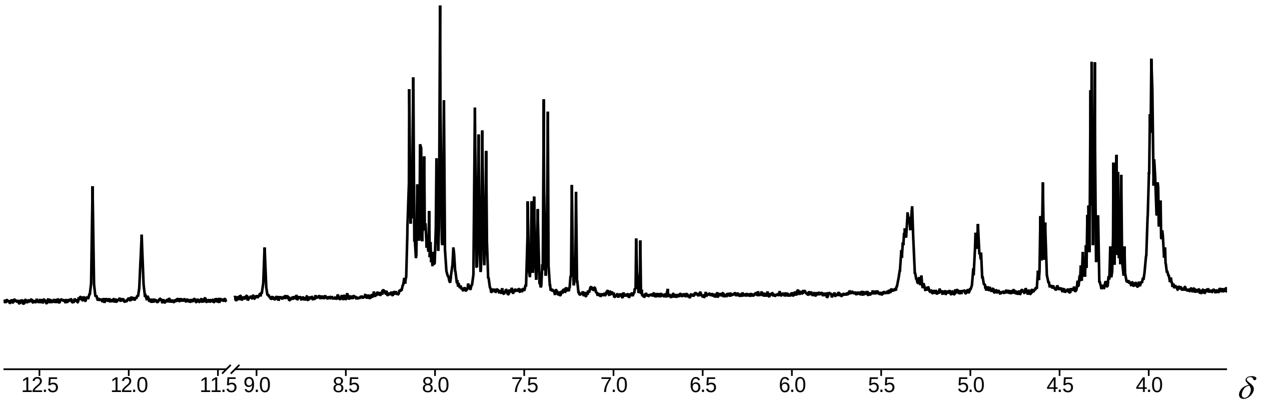

Figure S28: ^{1}H NMR spectra of the photostationary state of **1** after irradiation with 300 nm light in CD_3CN at 294K. The ratio of *Z*:*E* of the *para*-NO_2 and pentafluoro-functionalized hydrazones are 70:30 and 53:47, respectively.

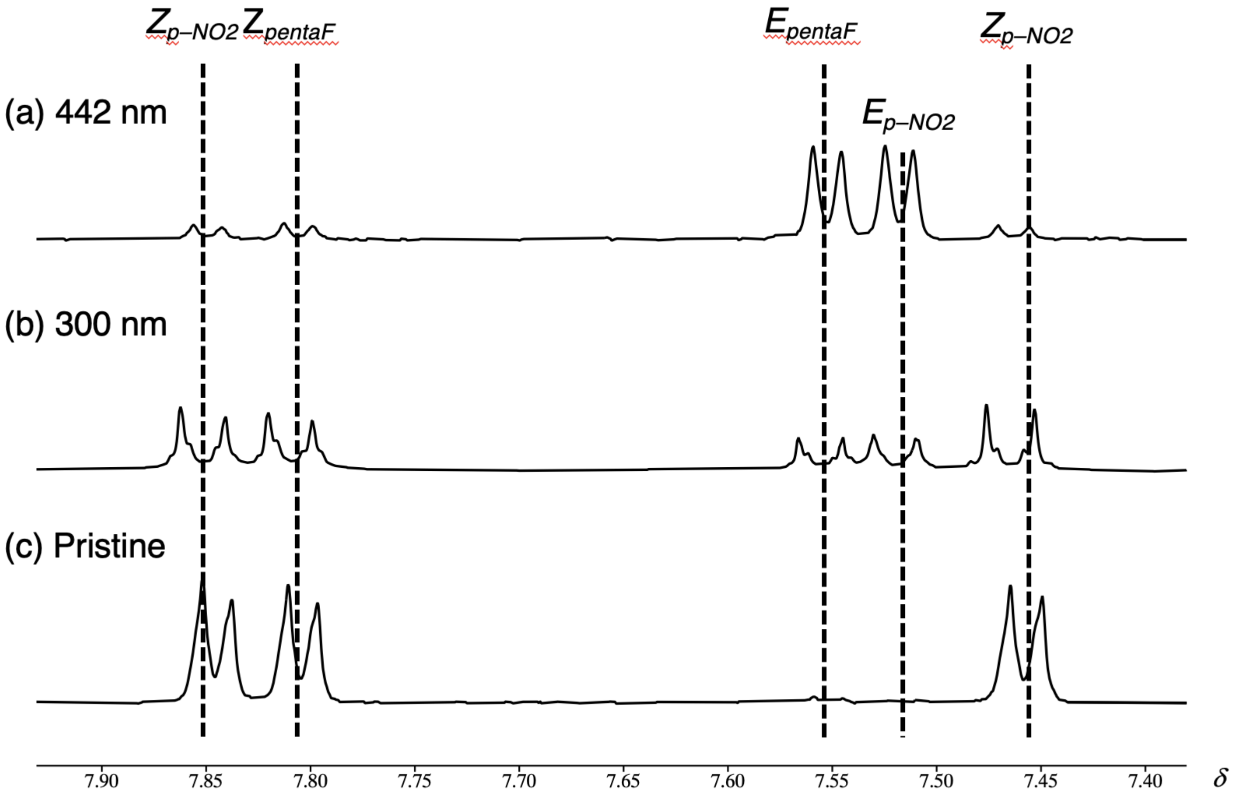

Figure S29: Zoom in on the ^{1}H NMR spectra of the (a) 442 nm, (b) 300 nm photostationary states and (c) pristine of **1** after sequential irradiation in CD_3CN at 294K.

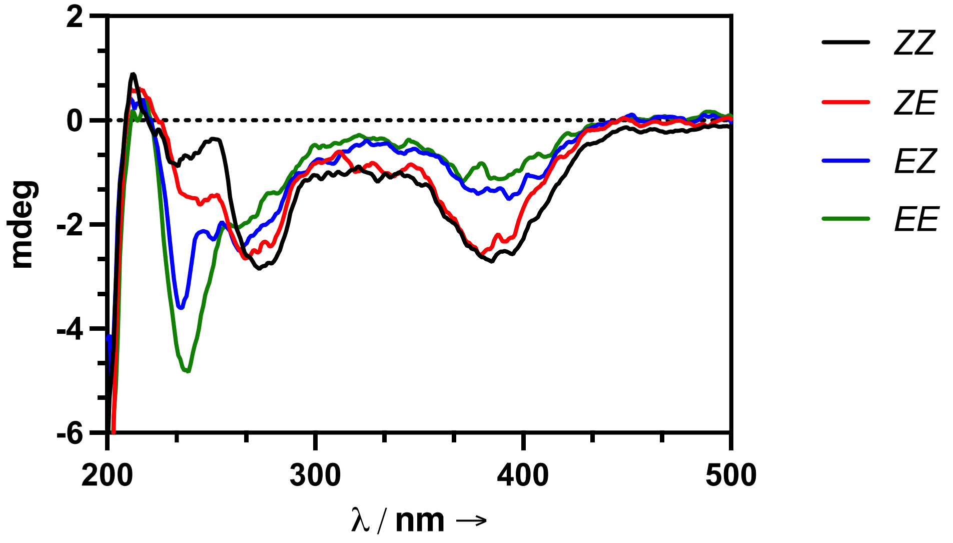

Figure S30: CD spectra in MeCN (2.5 × 10^{-4} M) of the *ZZ*, *ZE*, *EZ*, and *EE* isomer states of compound **1** upon sequential irradiation with 442, 340, and 442 nm light.

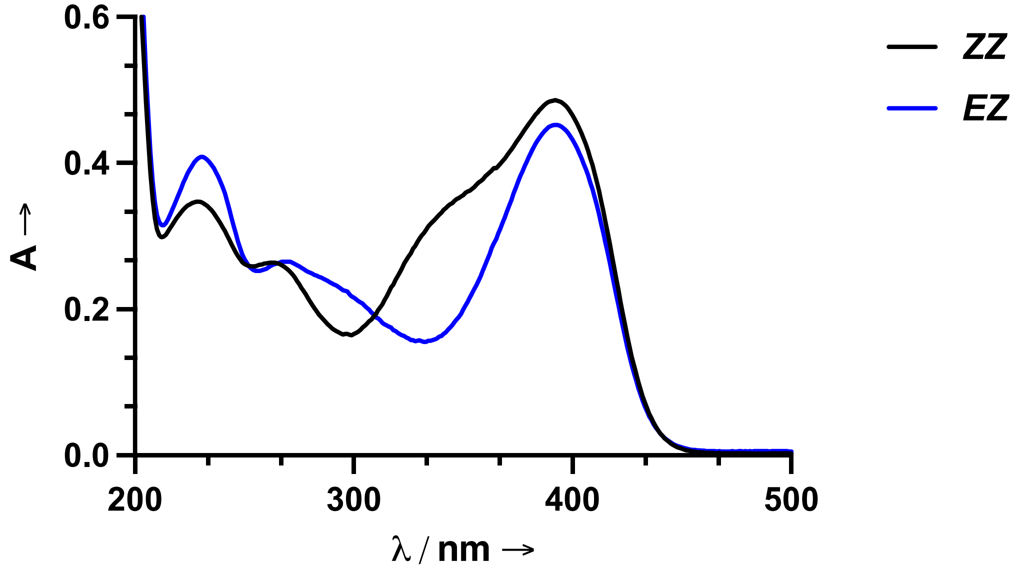

Figure S31: UV-Vis absorption spectra of **2**-*ZZ* and **2**-*EZ* in MeCN (1.0 x 10^{-5} M): The UV spectrum of **2**-*EZ* was obtained upon 340 nm light irradiation of the pristine sample.

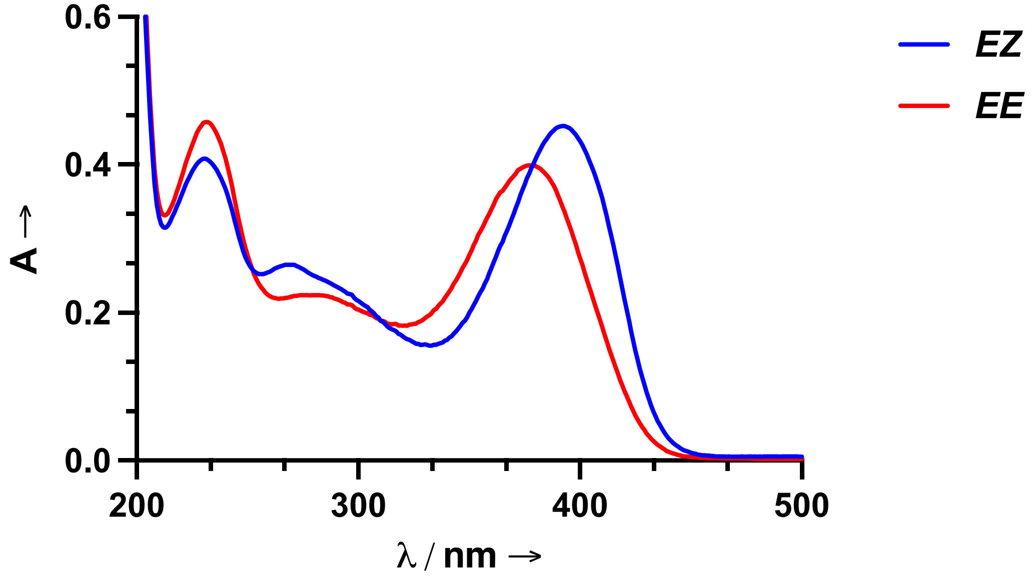

Figure S32: UV-Vis absorption spectra of **2**-*EZ* and **2**-*EE* in MeCN (1.0 x 10^{-5} M): The UV spectrum of **2**-*EE* was obtained upon 442 nm light irradiation of **2**-*EZ*.

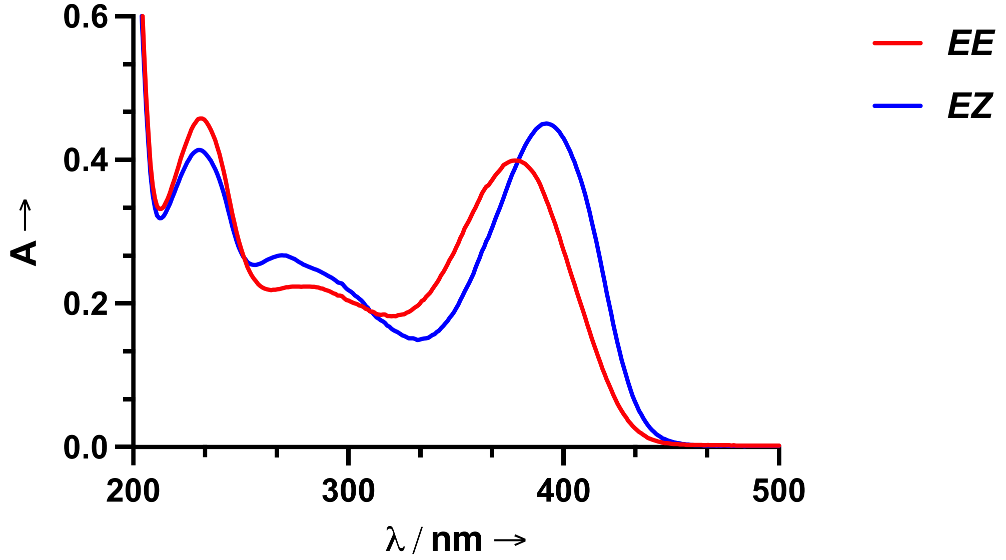

Figure S33: UV-Vis absorption spectra of **2**-*EE* and **2**-*EZ* in MeCN (1.0 x 10^{-5} M): The UV spectrum of **2**-*EZ* was obtained upon 340 nm light irradiation of **2**-*EE*.

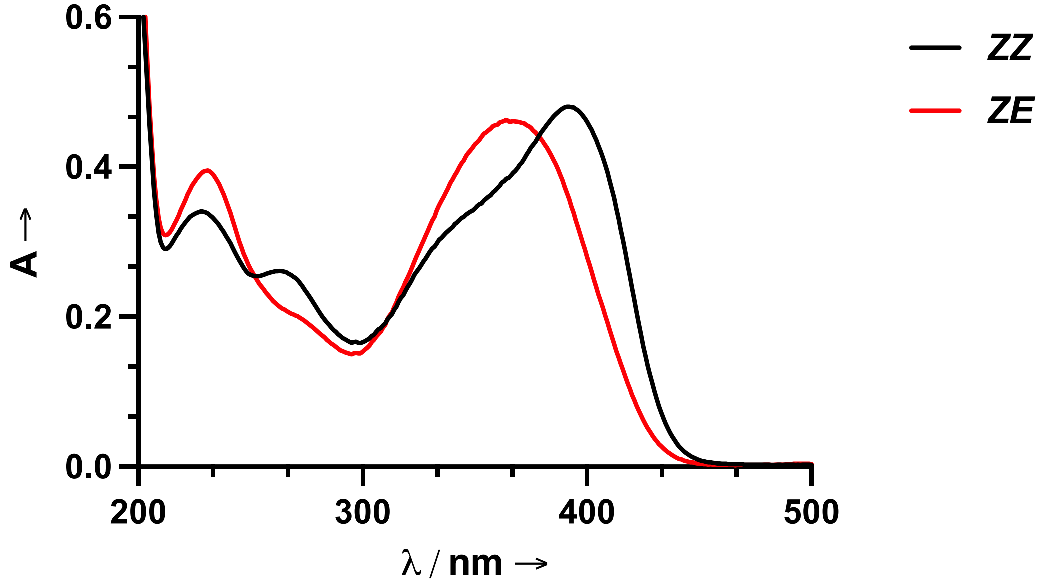

Figure S34: UV-Vis absorption spectra of **2**-Z*Z* and **2**-*ZE* in MeCN (1.0 x 10^{-5} M): The UV spectrum of **2**-*ZE* was obtained upon 442 nm light irradiation of the pristine sample.

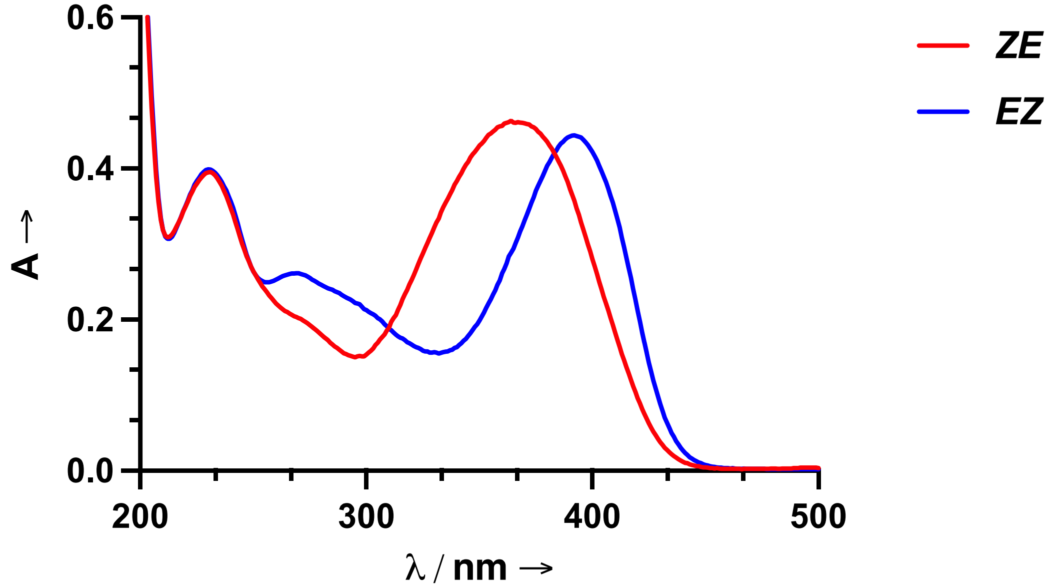

Figure S35: UV-Vis absorption spectra of **2**-*ZE* and **2**-*EZ* in MeCN (1.0 x 10^{-5} M): The UV spectrum of **2**-*EZ* was obtained upon 340 nm light irradiation of **2**-*ZE*.

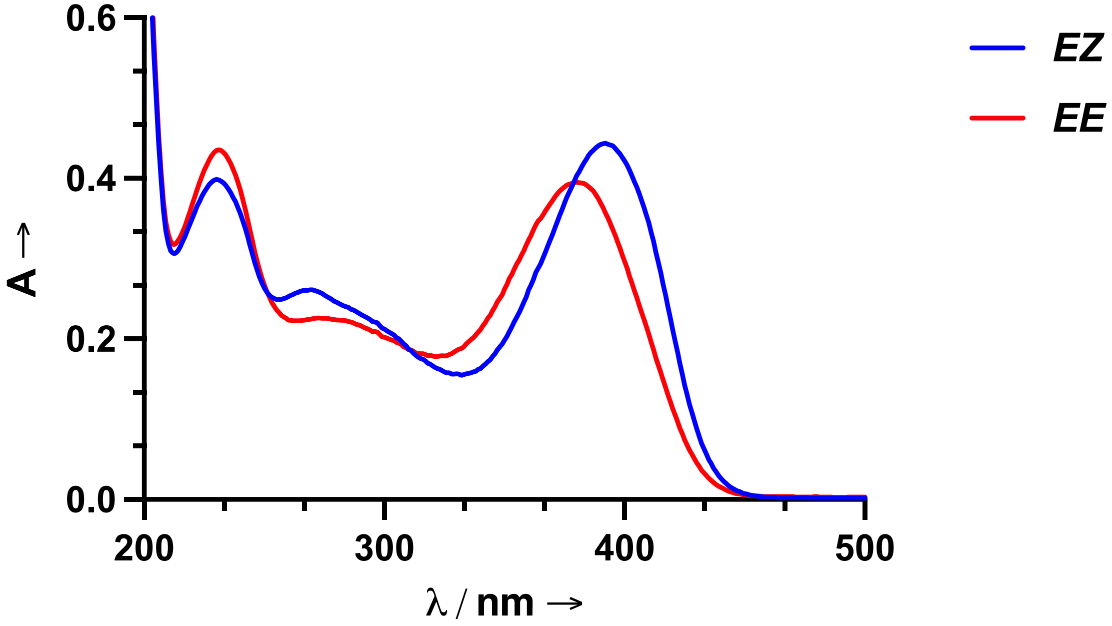

Figure S36: UV-Vis absorption spectra of **2**-*EZ* and **2**-*EE* in MeCN (1.0 x 10^{-5} M): The UV spectrum of **2**-*EE* was obtained upon 442 nm light irradiation of **2**-*EZ*.

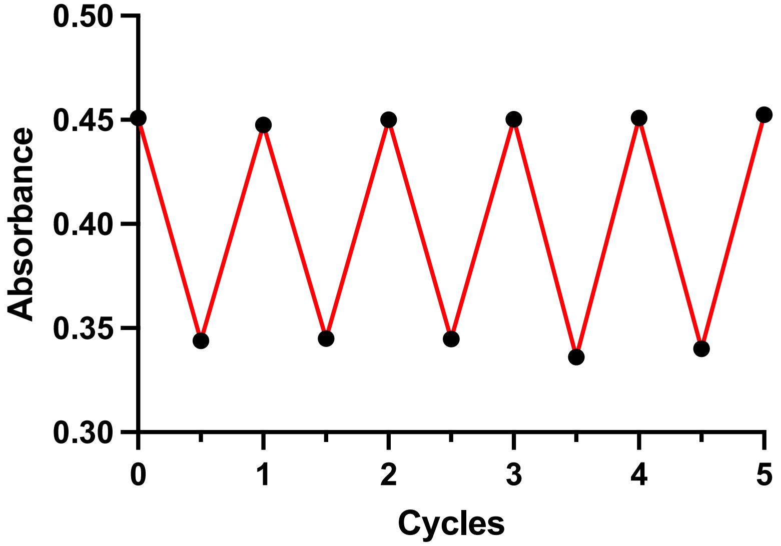

Figure S37: Photoisomerization cycles of hydrazone **2** in MeCN (1.0 × 10^{-5} M) at 294 K. The absorbance change at 392 nm was monitored while alternating the irradiation wavelength between 340 and 442 nm.

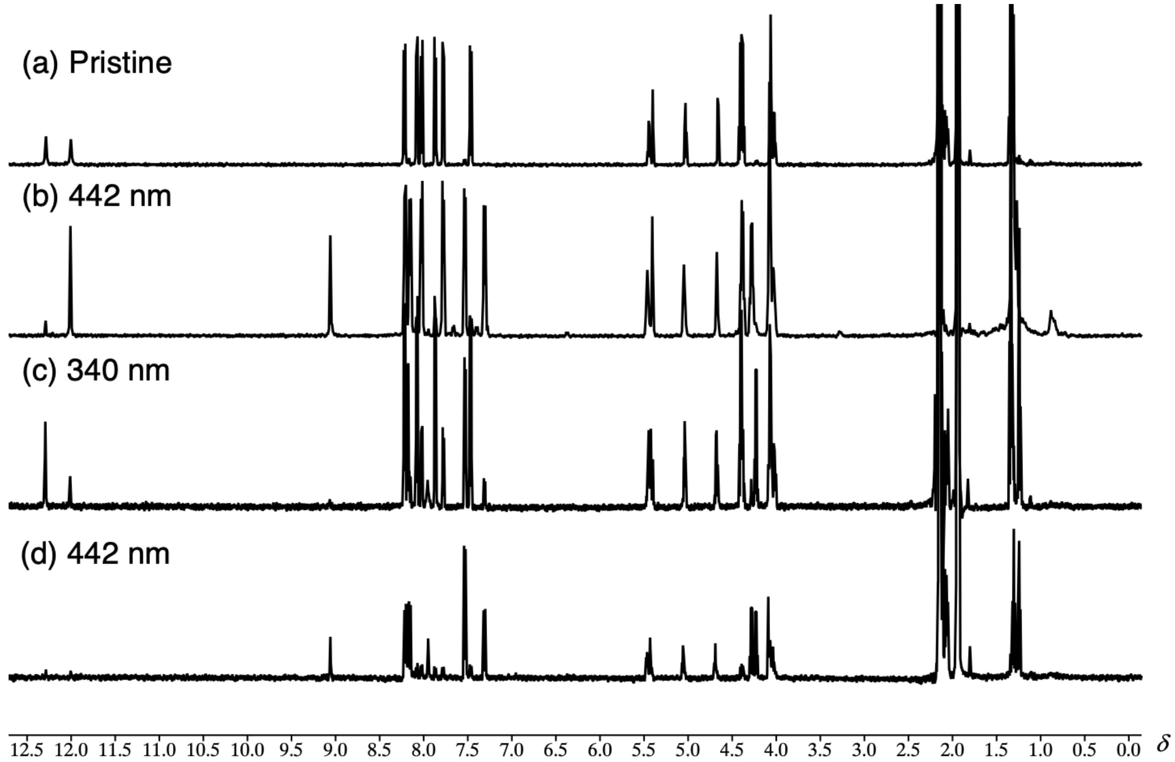

Figure S38: ^{1}H NMR spectra of (a) the pristine sample and the (b) 442, (c) 340, and (d) 442 nm photostationary states of **2** after sequential irradiation in CD_3CN at 294K.

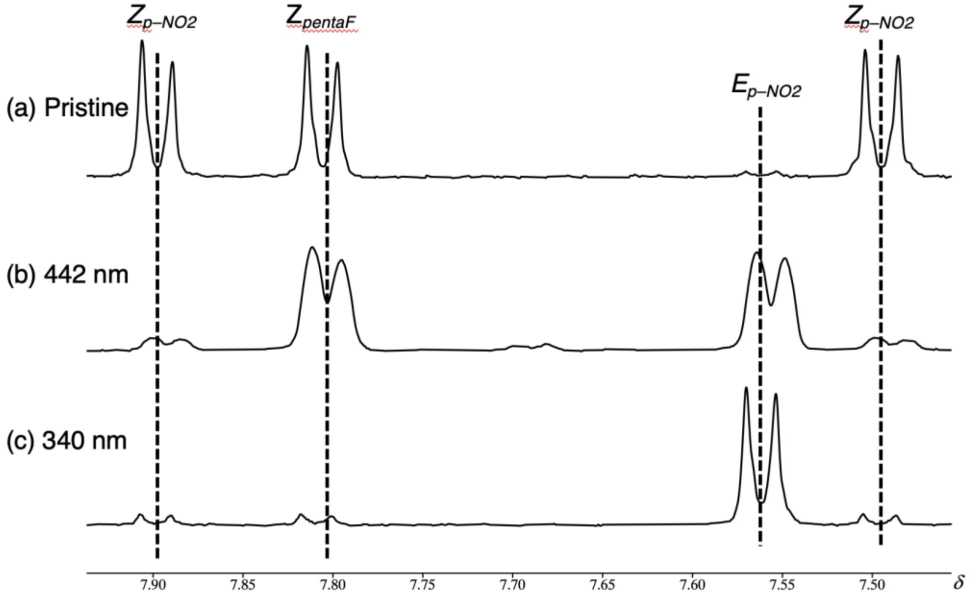

Figure S39: Zoom in on the ^{1}H NMR spectra of (a) the pristine sample and the (b) 442, (c) 340 nm photostationary states of **2** after sequential irradiation in CD_3CN at 294K.

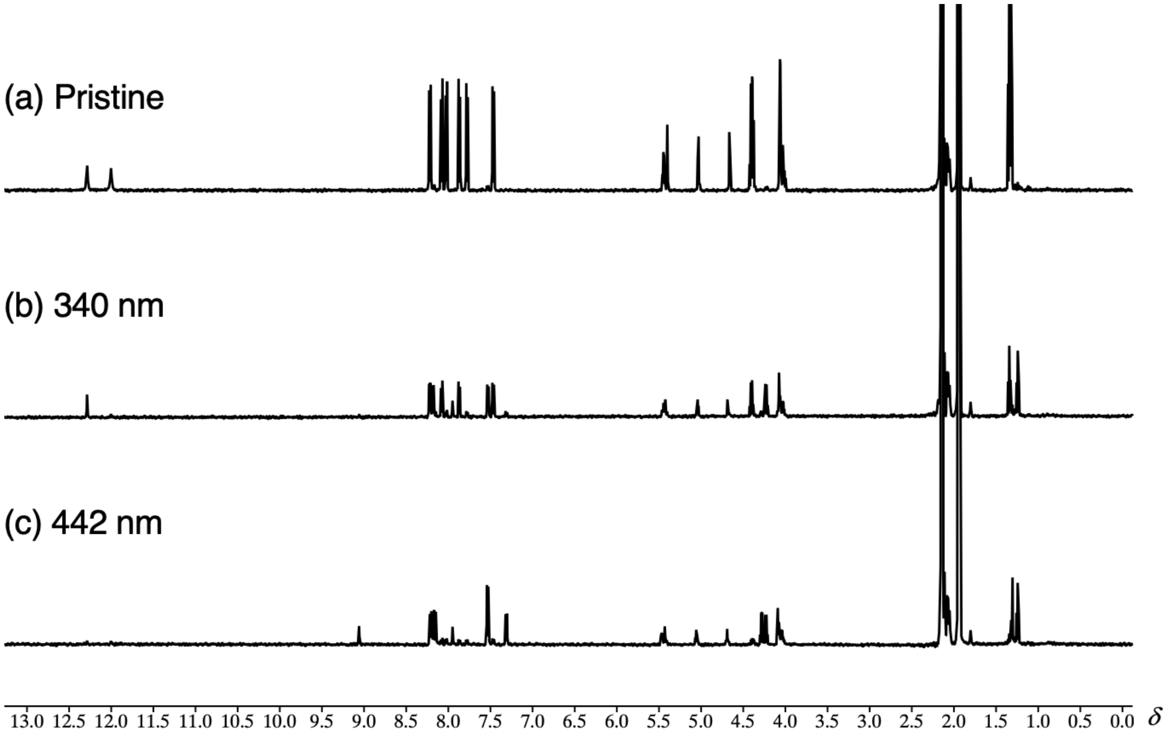

Figure S40: ^{1}H NMR spectra of (a) the pristine sample and the (b) 340, (c) 442 nm photostationary states of **2** after sequential irradiation in CD_3CN at 294K

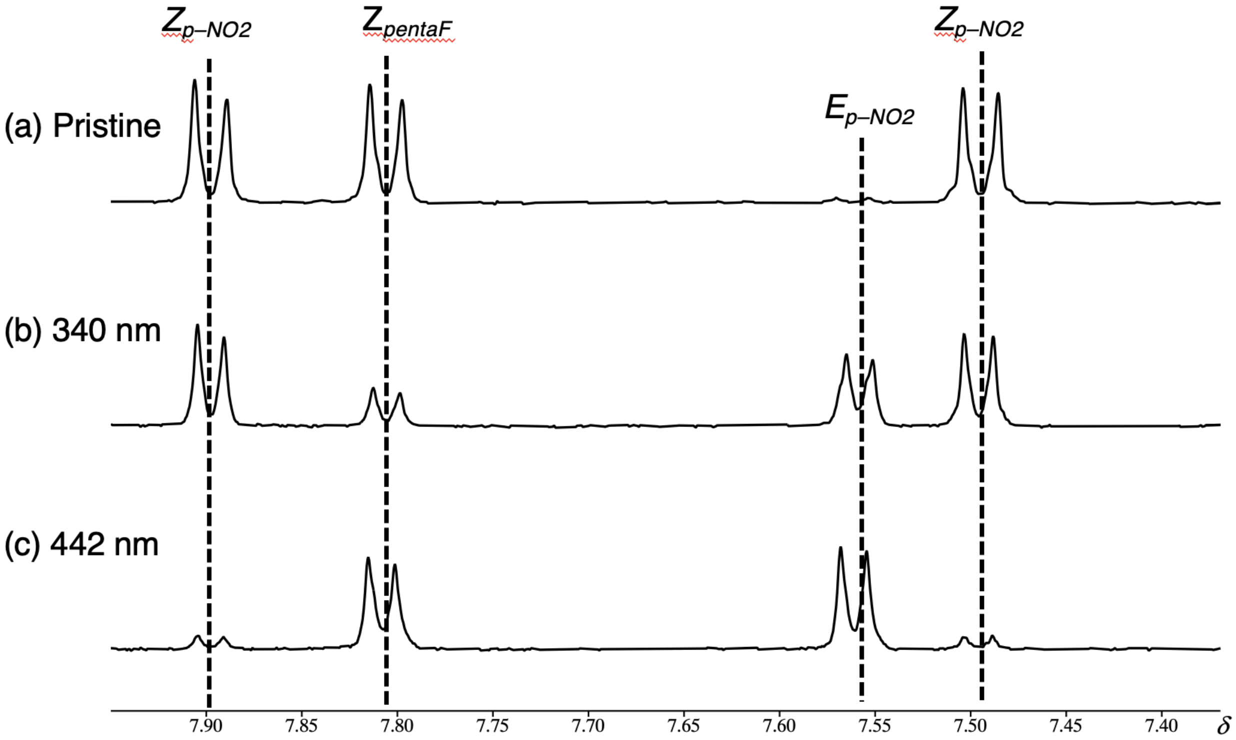

Figure S41: Zoom in on the ^{1}H NMR spectra of (a) the pristine sample and the (b) 340, (c) 442 nm photostationary states of **2** after sequential irradiation in CD_3CN at 294K.

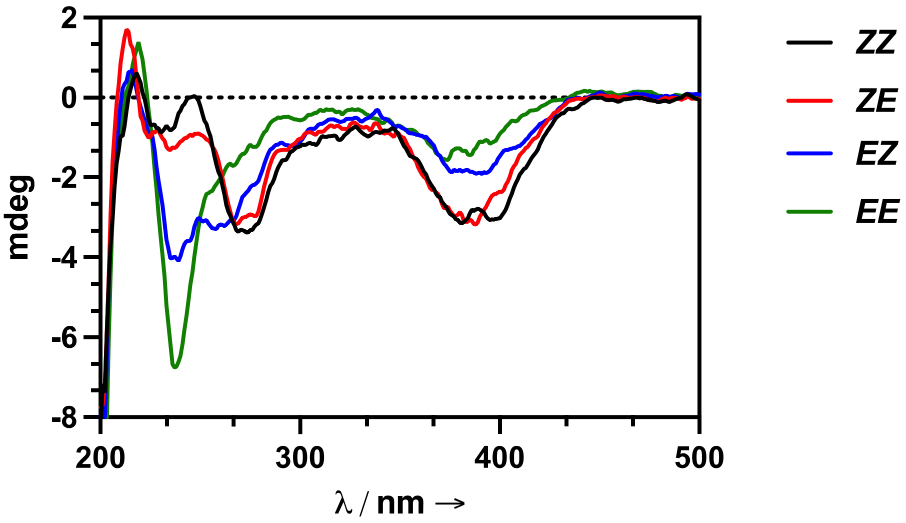

Figure S42: CD spectra in MeCN (2.5×10^{-4} M) of the *ZZ*, *ZE*, *EZ*, and *EE* isomer states of compound **2** upon sequential irradiation with 442, 340, and 442 nm light.

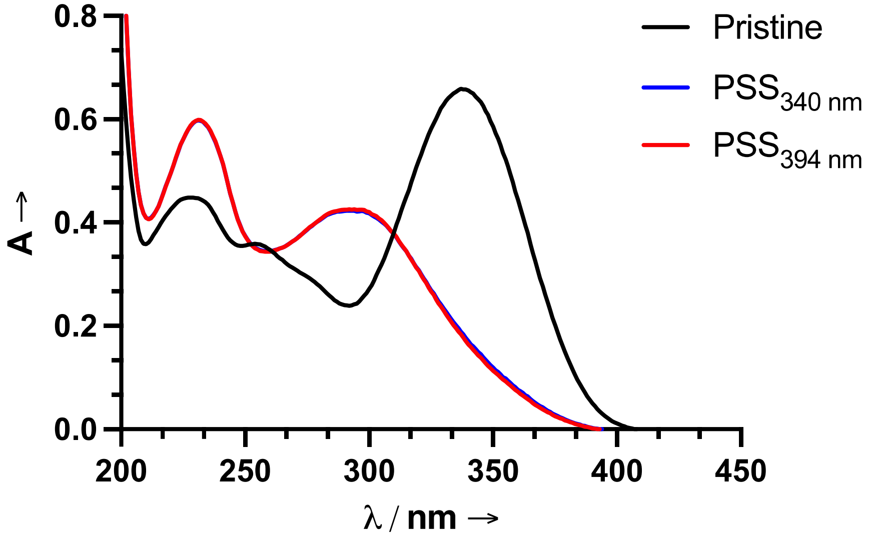

Figure S43: UV-Vis absorption spectra of **3** in MeCN (2.0 x 10^{-5} M): The UV spectrum of **3**-*EE* was obtained upon 340 nm light irradiation of **3**-*ZZ*. The UV spectrum of **3**-*EE* was also obtained upon 394 nm light irradiation.

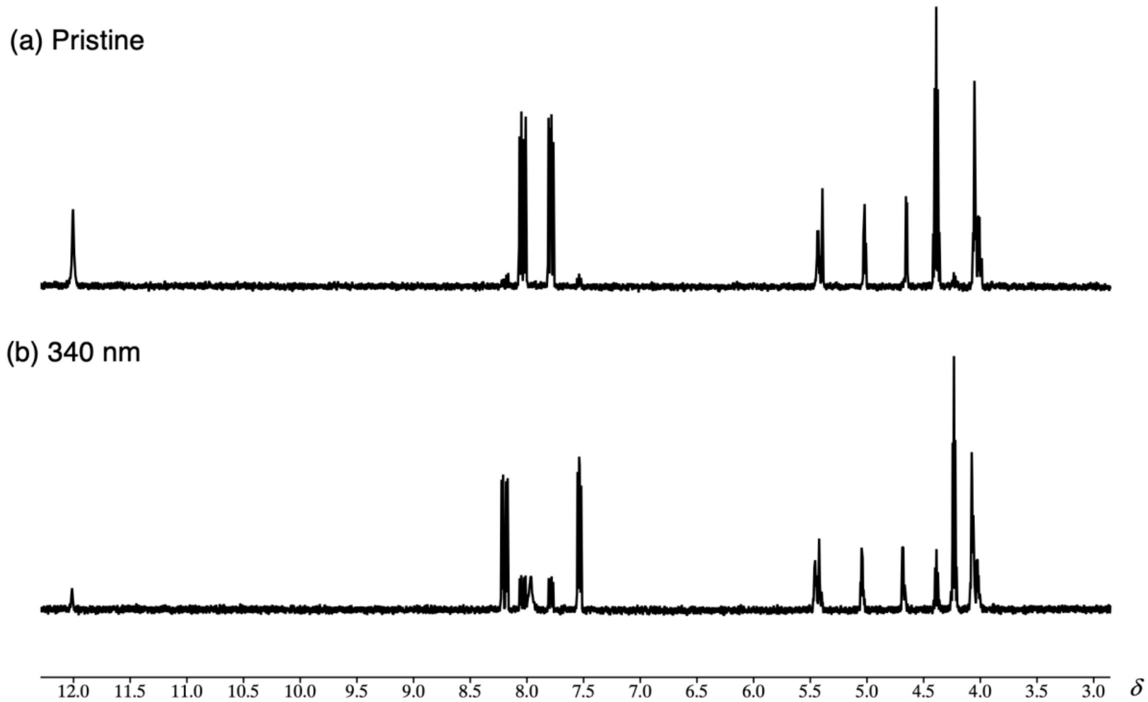

Figure S44: ^{1}H NMR spectra of (a) the pristine sample and the (b) 340 nm photostationary state of **3**.

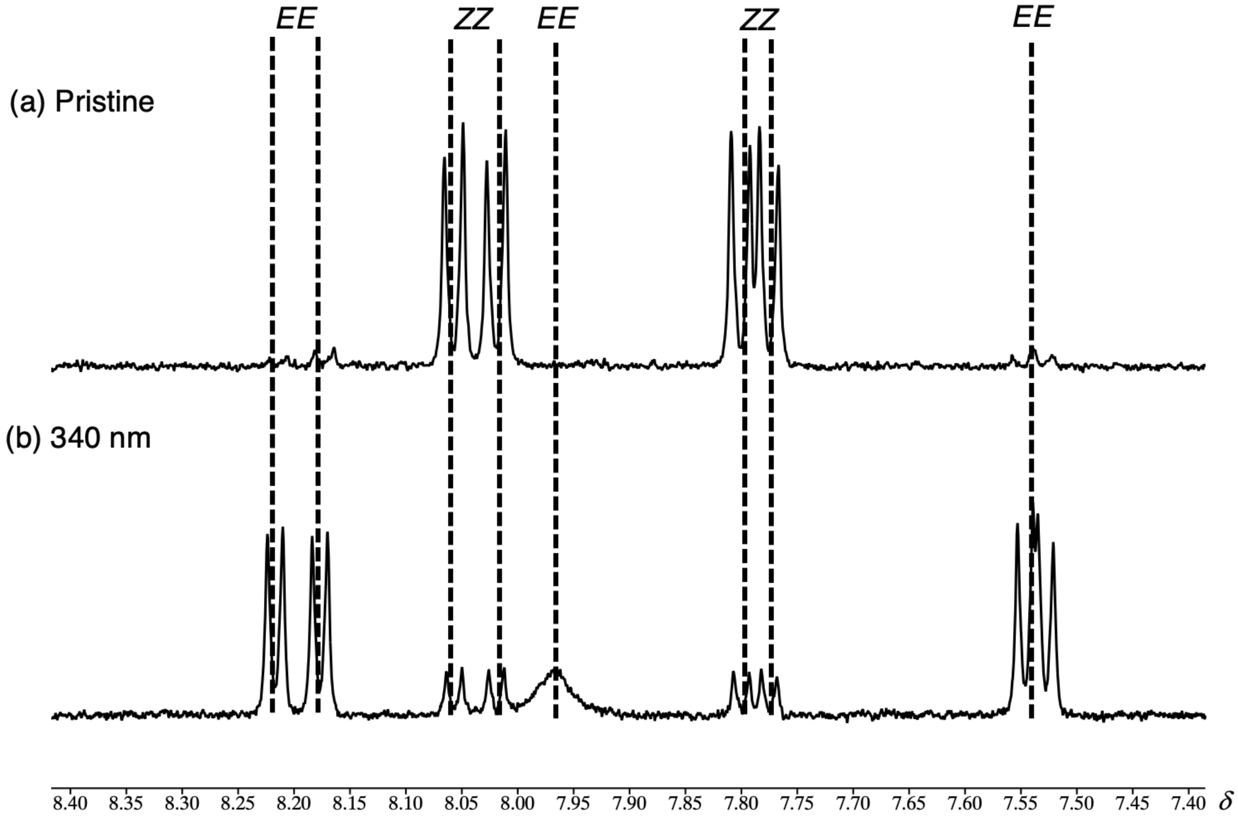

Figure S45: Zoom in on the ^{1}H NMR spectra of (a) the pristine sample and the (b) 340 nm photostationary state of **3** after irradiation in CD_3CN at 294K.

5. Determination of the Quantum Yield

$$A \underset{k_r}{\overset{k_f}{\rightleftharpoons}} B$$

In a photochemical reaction, species ***A*** absorbs light to generate product ***B***. The general kinetics of a basic photochemical reaction can be expressed using Eq. 1[S2]

$$r_{A\to B} = \frac{I_0 \Phi_{A\to B}}{V}\left(1 - 10^{-\varepsilon_A C_A l}\right) \qquad \text{Eq. 1}$$

When $\varepsilon_A \cdot C_A \cdot l \cdot \ln 10 << 1$ (or $Abs_A << 0.43$), the Taylor expansion can be truncated at the first-order term, simplifying Eq. 1 to Eq. 2, which is related to the quantum yield, an observed first-order rate constant, molar photon flux, and the measurable properties of sample ***A***.

$$k_{A\to B} = \frac{I_0 \Phi_{A\to B}}{V}\varepsilon_A \cdot l \cdot \ln 10 \qquad \text{Eq. 2}$$

Rearranging Eq. 2 gives Eq. 3,

$$\Phi_{A\to B} = \frac{k_{A\to B} V}{I_0 \varepsilon_A l \ln 10} \qquad \text{Eq. 3}$$

where $\Phi_{A\to B}$ is the photoisomerization yield going from ***A*** to ***B***; $k_{A\to B}$ represents the rate constant (obtained from the exponential fit of a graph of *Abs* vs. time); V indicates sample volume; I_0 indicates molar photo flux; ε_A indicates molar absorption coefficient of sample ***A***; and l indicates light path length.

The molar photon flux I_0 at 340, 394, and 442 nm were determined using chemical actinometry.[S3] A 0.002 L (= V_0) solution of potassium ferrioxalate in 0.05 M H_2SO_4 was placed in an 1.0 cm cuvette and irradiated for 30 s (= t_0). The irradiated solution was combined with 3.5 equiv. of ferrozine and stirred under dark for an hour. The resulting solution, containing reddish-purple $[Fe(ferrozine)_3]^{2+}$ complex was diluted by a factor of 30 (= n), and its absorbance was measured at 563 nm (A_{563}), where its molar absorption coefficient (ε_{563}) is 27,900 cm^{-1} M^{-1}. The molar photon flux I_0 of the light source at different wavelength was determined ($I_{0,340}$ = 7.76 x 10^{-8}, $I_{0,442}$ = 6.56 x 10^{-8} mol/s) using Eq. 4.

$$I_0\left(mol \cdot s^{-1}\right) = \frac{A_{563} \cdot n \cdot V_0}{\varepsilon_{563} \cdot l \cdot t_0 \cdot \phi_\lambda} \qquad \text{Eq. 4}$$

where l indicates the length of the cuvette, and ϕ_λ stands for the quantum yield of the photo-reduction of Fe(III) oxalate induced by the light source (ϕ_{340} = 1.25 and ϕ_{442} = 1.11).

The rate law for the formation of ***B*** is,

$$C_B = \frac{k_f}{k_f + k_r} C_{total} \left(1 - e^{-(k_f + k_r)t}\right) \qquad \text{Eq. 5}$$

where $C_{\text{total}} = C_A + C_B$ is the total concentration of the photoswitch and k_f and k_r are the first order approximate rate constants for the forward and reverse photochemical reactions under the low absorption approximation discussed above.

Rearranging Eq. 5 gives Eq. 6 that can be used to assess species ***A***,

$$C_A = \frac{C_{total} \cdot k_r}{k_f + k_r} + \frac{C_{total} \cdot k_f}{k_f + k_r} e^{-(k_f + k_r)t} \qquad \text{Eq. 6}$$

C_A is proportional to the absorbance of species ***A***. An exponential fit of absorbance as a function of time was performed to give the observed rate constant $k_{\text{obs}} = k_f + k_r$. For the new family photochromic hydrazone switches the thermal relaxation is extremely slow at room temperature, and so it is not considered during the calculation. Thus, $k_{A \rightarrow B} \approx k_{\text{obs}}$

Table S1: Photophysical data for compound **1**.

**1**		**λ_{max} (nm) / ε**	***Φ***	**PSS**[a]
**Pristine**[b]	*ZZ*	388 / 46300	---	99:1; 99:1
**340 nm**	*EZ*	392 / 45500	9.2 ± 0.7%	12:88; 89:11
**442 nm**	*EE*	379 / 40500	9.9 ± 0.4%	14:86; 14:86
**340 nm**	*EZ*	392 / 45900	20.7 ± 2.8%	13:87; 88:12
**Pristine**[c]	*ZZ*	388 / 46300	---	99:1; 99:1
**442 nm**	*ZE*	364 / 45600	14.6 ± 2.1%	87:13; 13:87
**340 nm**	*EZ*	392 / 44500	11.5 ± 0.3%	15:85; 88:12
**442 nm**	*EE*	380 / 38800	9.9 ± 0.4%	12:88; 13:87

a PSS values are the ratio of *Z*/*E* of pentafluoro and *para*-NO_2 hydrazones, respectively measured in MeCN.
b Quantum yields were determined after sequential irradiation of the pristine with 340, 442, and 340 nm light.
c Quantum yields were determined after sequential irradiation of the pristine with 442, 340, and 442 nm light.

Table S2: Photophysical data for compound **2**.

**2**		**λ_{max} (nm) / ε**	***Φ***	**PSS[a]**
**Pristine[b]**	*ZZ*	392 / 48600	---	99:1; 99:1
**340 nm**	*EZ*	392 / 45200	10.8 ± 1.8%	16:84; 88:12
**442 nm**	*EE*	377 / 39900	8.7 ± 0.5%	14:86; 12:88
**340 nm**	*EZ*	392 / 45100	21.3 ± 0.9%	16:84; 88:12
**Pristine[c]**	*ZZ*	392 / 48600	---	99:1; 99:1
**442 nm**	*ZE*	367 / 46100	8.3 ± 0.3%	95:5; 11:89
**340 nm**	*EZ*	392 / 44400	10.7 ± 0.4%	13:87; 89:11
**442 nm**	*EE*	380 / 39500	8.7 ± 0.5%	14:86; 12:88

a PSS values are the ratio of *Z*/*E* of pentafluoro and *para*-NO_2 hydrazones, respectively measured in MeCN.
b Quantum yields were determined after sequential irradiation of the pristine with 340, 442, and 340 nm light.
c Quantum yields were determined after sequential irradiation of the pristine with 442, 340, and 442 nm light.

Table S3: Photophysical data for compounds **3** and **4**.

	**$\lambda_{max,Z}$ (nm) / ε**	**$\lambda_{max,E}$ (nm) / ε**	**$\Phi_{Z\rightarrow E}$**	**$\Phi_{E\rightarrow Z}$**	**PSS_{340nm}**	**PSS_{442nm}**
**3**	338 / 48100	298 / 28500	7.7± 0.5%	---[a]	90:10	---[a]
**4[b]**	394 / 59900	368 / 50600	17.4 ± 0.6%	8.6 ± 0.9%	89:11	1:99

a EE$\rightarrow$*ZZ* photoisomerization was not measured.
b See publication[S1] for more details on photophysical characterization of compound **4**.

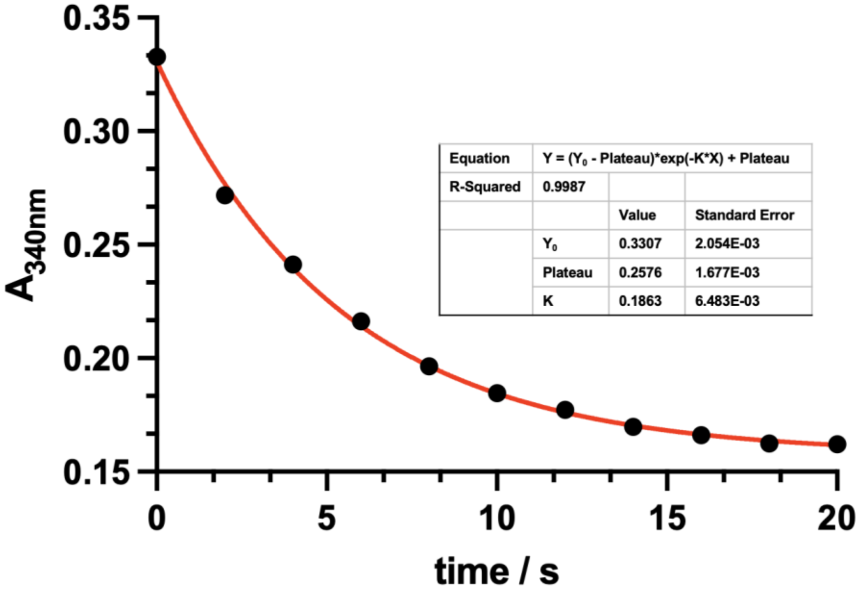

Figure S46: Kinetics of the photoisomerization from the pristine state of **1**-*ZZ* to **1**-*EZ* in MeCN (1.0×10^{-5} M) upon irradiation with 340 nm light at 298 K; the plot is of the absorbance (λ_{max} = 340 nm) of **1**-*EZ* as a function of time. $\varepsilon_{\mathbf{1}\text{-}ZZ@340nm}$ = 33,300 $M^{-1}\cdot cm^{-1}$ was used for quantum yield calculations. The photoisomerization quantum yield was calculated to be 9.2 ± 0.7% based on three consecutive measurements.

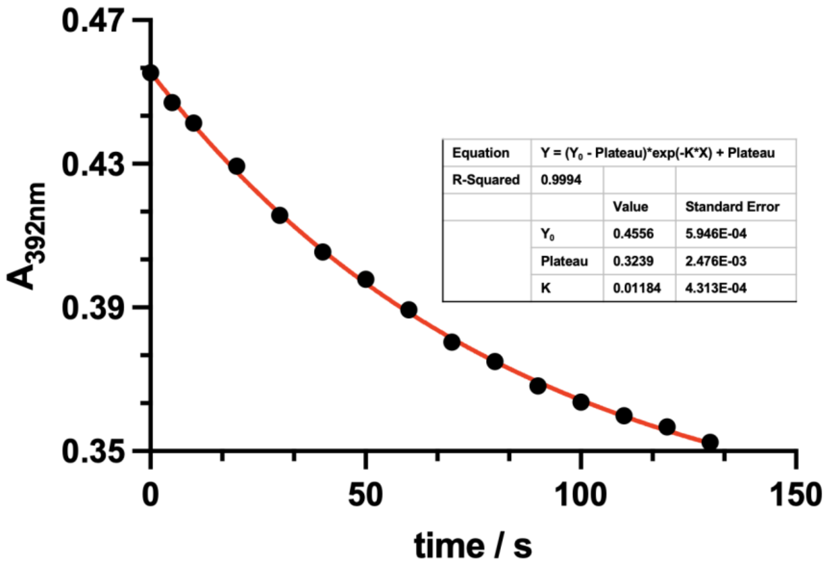

Figure S47: Kinetics of the photoisomerization from the PSS_{340} of **1**-*EZ* to **1**-*EE* in MeCN (1.0×10^{-5} M) upon irradiation with 442 nm light at 298 K; the plot is of the absorbance (λ_{max} = 392 nm) of **1**-*EE* as a function of time. $\varepsilon_{\mathbf{1}\text{-}EZ@442nm}$ = 2,260 $M^{-1}\cdot cm^{-1}$ was used for quantum yield calculations. The photoisomerization quantum yield was calculated to be 9.9 ± 0.4% based on three consecutive measurements.

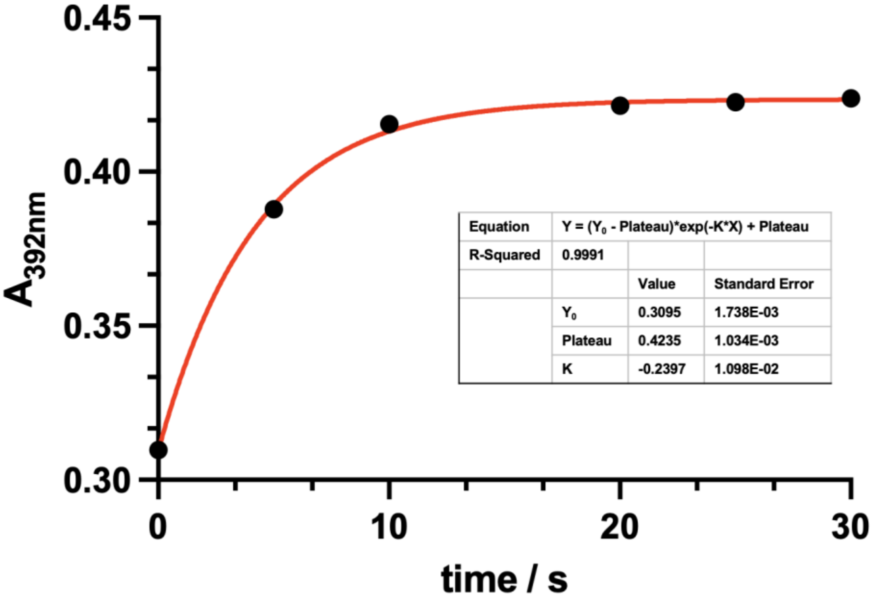

Figure S48: Kinetics of the photoisomerization from the PSS_{442} of **1**-*EE* to **1**-*EZ* in MeCN (1.0×10^{-5} M) upon irradiation with 340 nm light at 298 K; the plot is of the absorbance (λ_{max} = 392 nm) of **1**-*EZ* as a function of time. $\varepsilon_{\mathbf{1}\text{-}EE@340nm}$ = 22,000 $M^{-1}\cdot cm^{-1}$ was used for quantum yield calculations. The photoisomerization quantum yield was calculated to be 20.7 ± 2.7% based on three consecutive measurements.

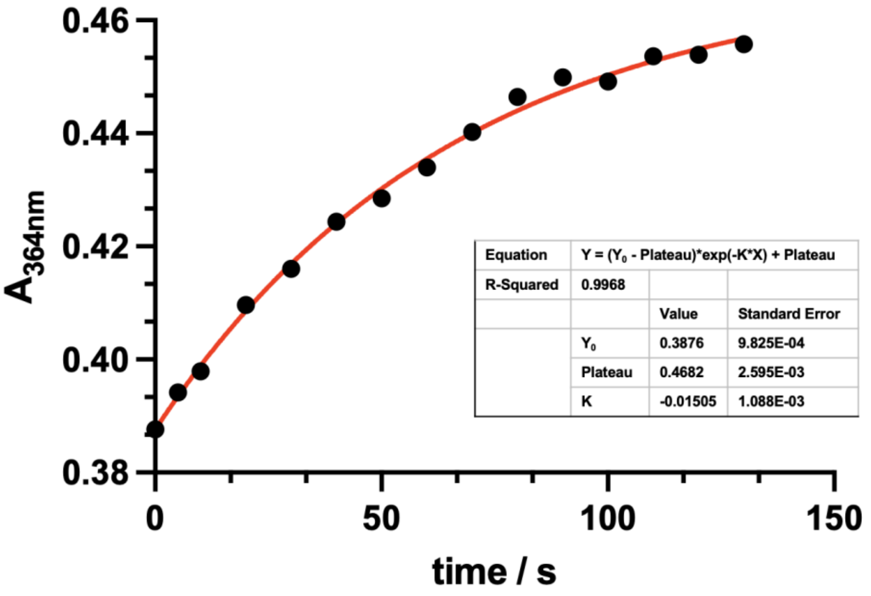

Figure S49: Kinetics of the photoisomerization from the pristine state of **1**-*ZZ* to **1**-*ZE* in MeCN (1.0×10^{-5} M) upon irradiation with 442 nm light at 298 K; the plot is of the absorbance (λ_{max} = 364 nm) of **1**-*ZE* as a function of time. $\varepsilon_{\mathbf{1}\text{-}ZZ@442nm}$ = 1,730 $M^{-1}\cdot cm^{-1}$ was used for quantum yield calculations. The photoisomerization quantum yield was calculated to be 14.6 ± 2.1% based on three consecutive measurements.

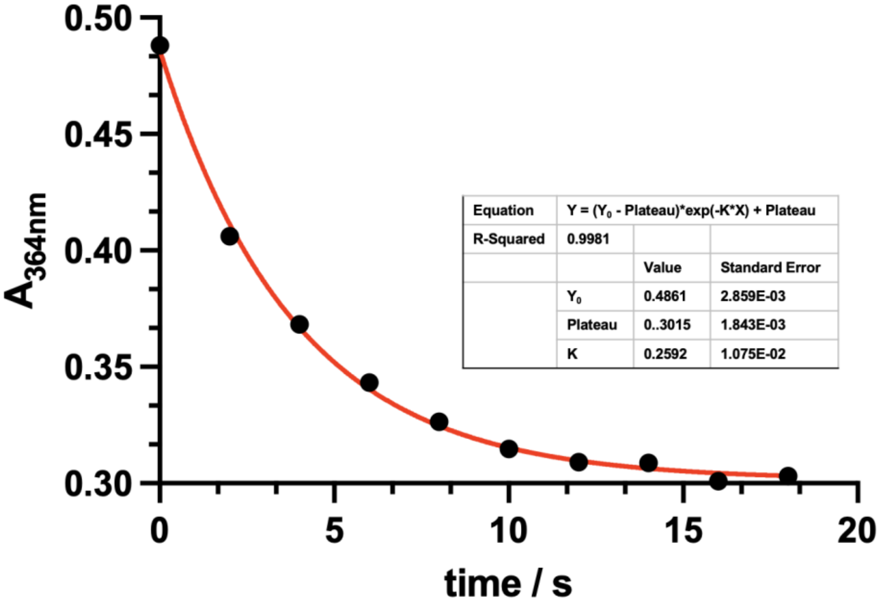

Figure S50: Kinetics of the photoisomerization from PSS_{442} of **1**-*ZE* to **1**-*EZ* in MeCN (1.0×10^{-5} M) upon irradiation with 340 nm light at 298 K; the plot is of the absorbance (λ_{max} = 364 nm) of **1**-*EZ* as a function of time. $\varepsilon_{1\text{-}ZE@340nm}$ = 37,300 $M^{-1}{\cdot}cm^{-1}$ was used for quantum yield calculations. The photoisomerization quantum yield was calculated to be 11.5 ± 0.3% based on three consecutive measurements.

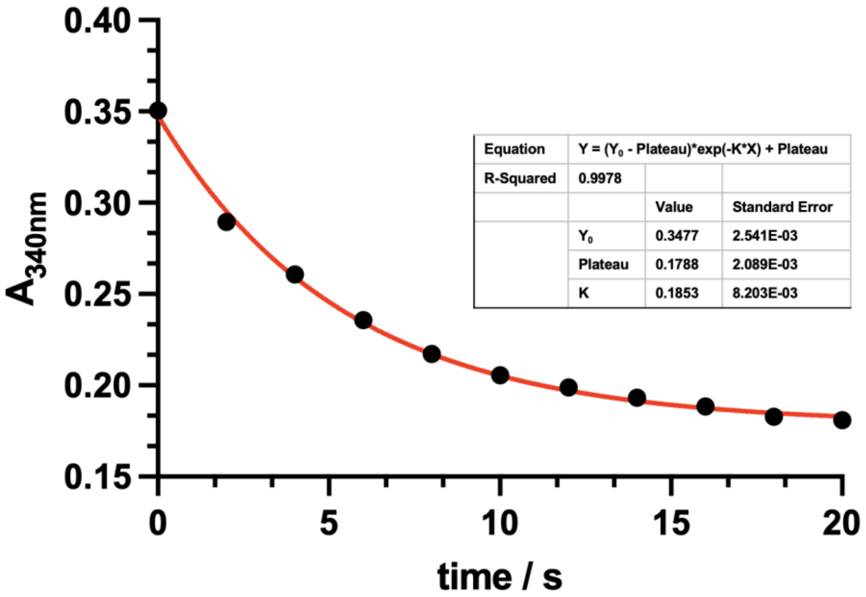

Figure S51: Kinetics of the photoisomerization from the pristine state of **2**-*ZZ* to **2**-*EZ* in MeCN (1.0×10^{-5} M) upon irradiation with 340 nm light at 298 K; the plot is of the absorbance (λ_{max} = 340 nm) of **2**-*EZ* as a function of time. $\varepsilon_{2\text{-}ZZ@340nm}$ = 33,200 $M^{-1}{\cdot}cm^{-1}$ was used for quantum yield calculations. The photoisomerization quantum yield was calculated to be 10.8 ± 1.8% based on three consecutive measurements.

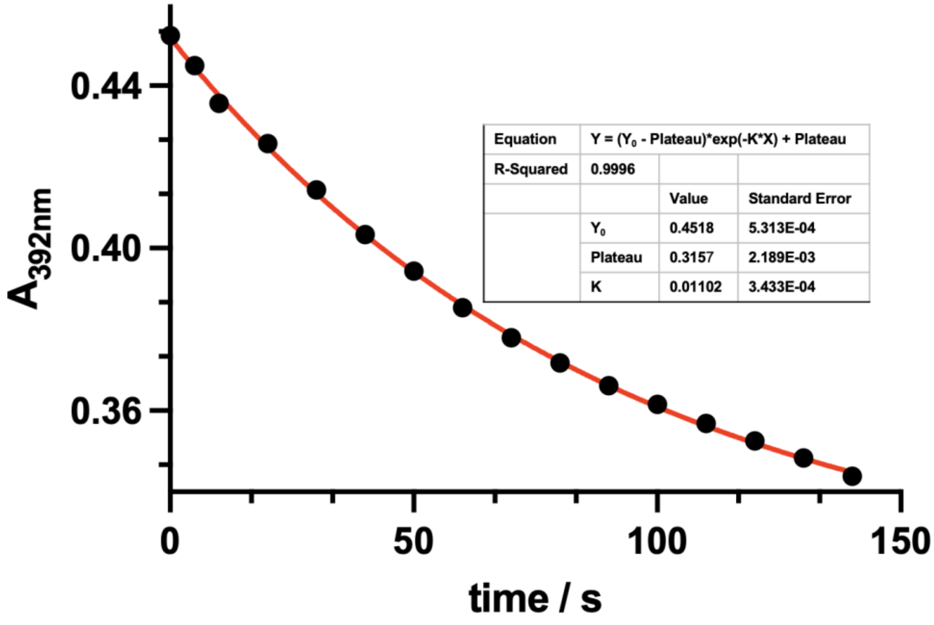

Figure S52: Kinetics of the photoisomerization from the PSS_{340} of **2**-*EZ* to **2**-*EE* in MeCN (1.0 × 10^{-5} M) upon irradiation with 442 nm light at 298 K; the plot is of the absorbance (λ_{max} = 392 nm) of **2**-*EE* as a function of time. $\varepsilon_{2\text{-}EZ@442nm}$ = 2,380 $M^{-1}\cdot cm^{-1}$ was used for quantum yield calculations. The photoisomerization quantum yield was calculated to be 8.7 ± 0.5% based on three consecutive measurements.

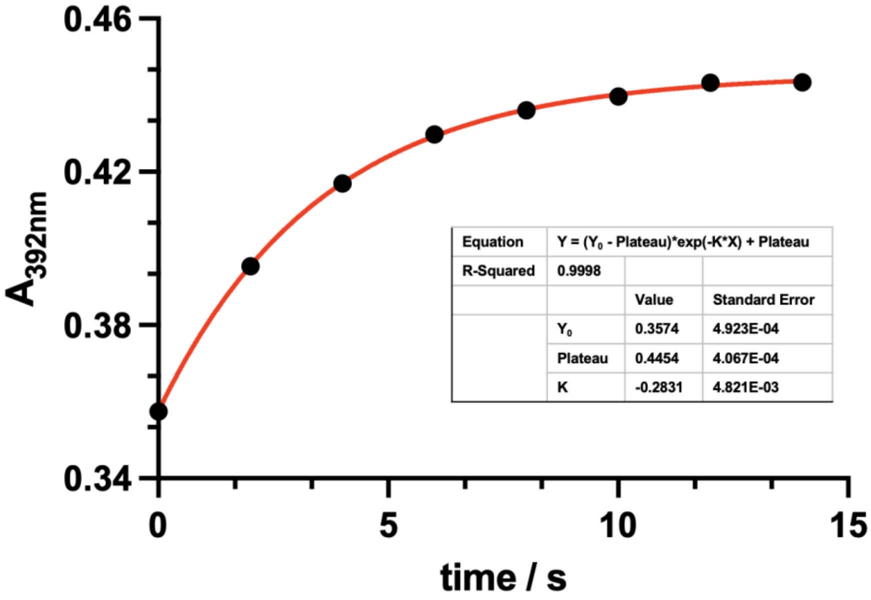

Figure S53: Kinetics of the photoisomerization from the PSS_{442} of **2**-*EE* to **2**-*EZ* in MeCN (1.0 × 10^{-5} M) upon irradiation with 340 nm light at 298 K; the plot is of the absorbance (λ_{max} = 392 nm) of **2**-*EZ* as a function of time. $\varepsilon_{2\text{-}EE@340nm}$ = 22,400 $M^{-1}\cdot cm^{-1}$ was used for quantum yield calculations. The photoisomerization quantum yield was calculated to be 21.3 ± 0.9% based on three consecutive measurements.

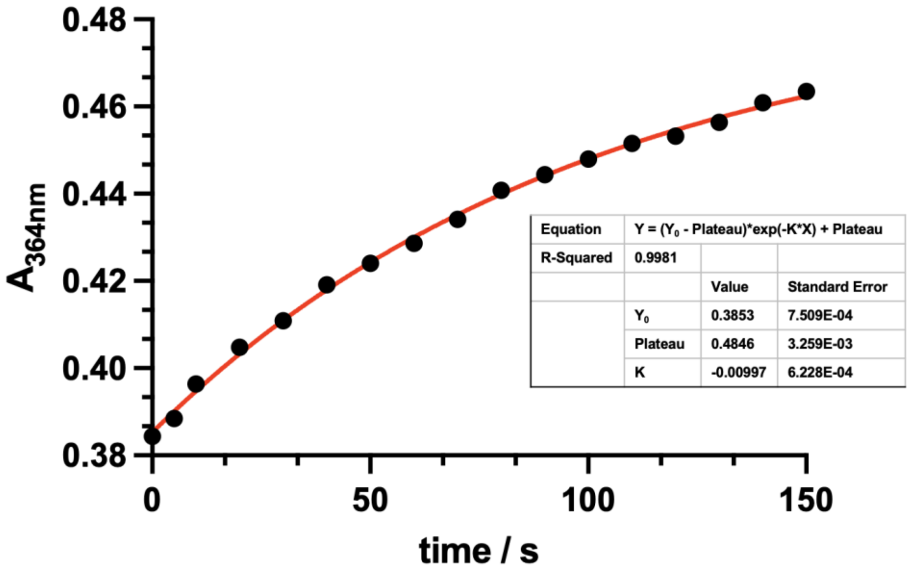

Figure S54: Kinetics of the photoisomerization from the pristine state of **2**-*ZZ* to **2**-*ZE* in MeCN (1.0 × 10^{-5} M) upon irradiation with 442 nm light at 298 K; the plot is of the absorbance (λ_{max} = 364 nm) of **2**-*ZE* as a function of time. $\varepsilon_{2\text{-}ZZ@442nm}$ = 2,390 $M^{-1}\cdot cm^{-1}$ was used for quantum yield calculations. The photoisomerization quantum yield was calculated to be 8.3 ± 0.3% based on three consecutive measurements.

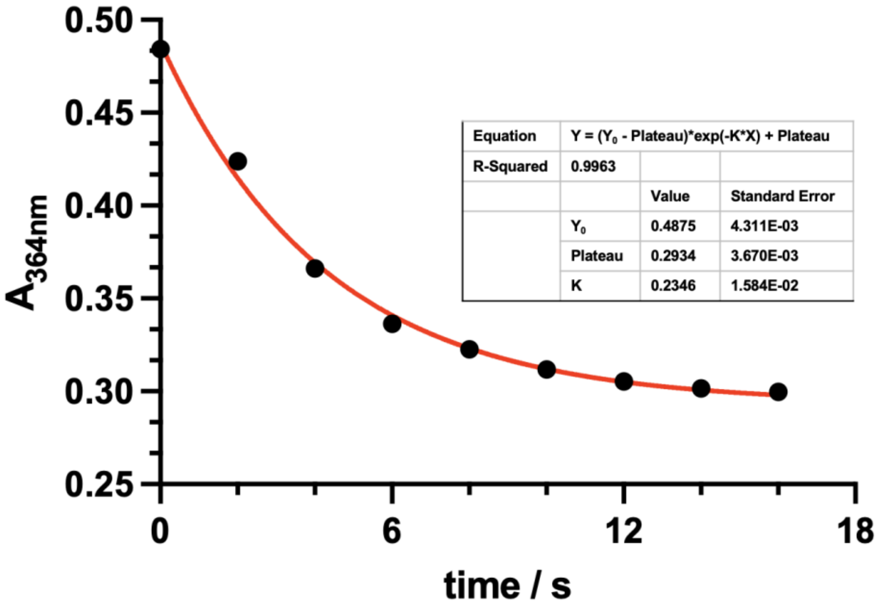

Figure S55: Kinetics of the photoisomerization from the PSS_{442} of **2**-*ZE* to **2**-*EZ* in MeCN (1.0 × 10^{-5} M) upon irradiation with 340 nm light at 298 K; the plot is of the absorbance (λ_{max} = 364 nm) of **2**-*EZ* as a function of time. $\varepsilon_{1\text{-}ZE@340nm}$ = 38,300 $M^{-1}\cdot cm^{-1}$ was used for quantum yield calculations. The photoisomerization quantum yield was calculated to be 10.7 ± 0.4% based on three consecutive measurements.

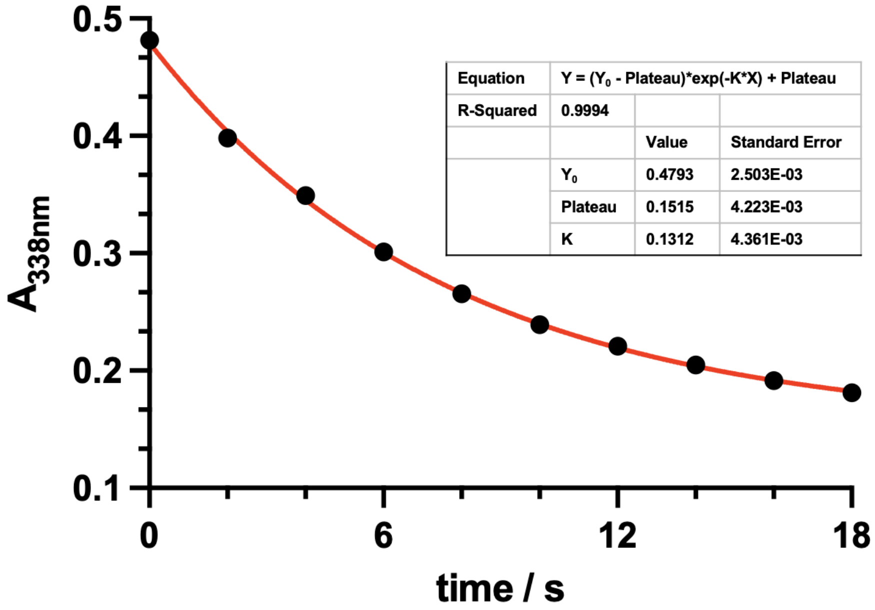

Figure S56: Kinetics of the photoisomerization from the pristine of **3**-*ZZ* to **3**-*EE* in MeCN (1.0×10^{-5} M) upon irradiation with 340 nm light at 298 K; the plot is of the absorbance (λ_{max} = 338 nm) of **3**-*ZZ* as a function of time. $\varepsilon_{\mathbf{1}\text{-}ZZ@340nm}$ = 48,000 $M^{-1}\cdot cm^{-1}$ was used for quantum yield calculations. The photoisomerization quantum yield was calculated to be 7.7 ± 0.5% based on three consecutive measurements.

6. Determination of the Thermal Isomerization Half-Life

The thermal isomerization kinetics of the hydrazones were studied at using ^{1}H NMR spectroscopy. Solutions (~10^{-3} M) of the hydrazone switches in 0.5 mL MeCN-d_3 were irradiated at a specific wavelength and then left in a Huber Kiss 212B circulating water bath at a preset temperature of 350K. ^{1}H NMR spectra were then acquired at different intervals at room temperature to monitor the change in aromatic signals at 7.98 and 7.55 ppm and 7.80 and 7.50 ppm. Integration of the hydrazone NH proton signal intensities gave the same changes as a function of time. The thermal isomerization rates (k_1) were determined by least-square curve fittings using an integrated and combined rate equation (Eq. 7) of a single-species reversible reaction:[S4]

$$\mathbf{A} \underset{k_{-1}}{\overset{k_1}{\rightleftharpoons}} \mathbf{Z}$$

$$K_{eq} = \frac{k_1}{k_{-1}} = \frac{C_A^0 - C_A^{eq}}{C_A^{eq}} \qquad \text{Eq. 7}$$

$$\ln\left(\frac{C_A - C_A^{eq}}{C_A^0 - C_A^{eq}}\right) = -(k_1 + k_{-1})t \qquad \text{Eq. 8}$$

Combining Eq. 7 and 8 will give Eq. 9 as follow,

$$C_A = \left(C_A^0 - C_A^{eq}\right) \times e^{\left(\frac{-k_1 \cdot C_A^0 \cdot t}{C_A^0 - C_A^{eq}}\right)} + C_A^{eq} \qquad \text{Eq. 9}$$

where C_A, C_A^0, and C_A^{eq} stand for experimental, initial, and equilibrium concentrations of the metastable configuration of the hydrazone switch, respectively; t stands for the thermal relaxation time.

The resulting k_1 values for the hydrazones at elevated temperatures were measured and then used to calculate the energy barriers for the $E \rightarrow Z$ thermal relaxation using the Eyring equation (Eq. 10.). With the energy barriers ($\Delta G^{\ddagger}$) in hand, the Arrhenius equation (Eq. 11) was used to extrapolate the rate constants (k_2) to 298K, from which the room-temperature thermal half-lives were determined.[S5]

$$\Delta G^{\ddagger} = 8.314 \times T \times [23.760 + \ln(\frac{T}{k})] \qquad \text{Eq. 10}$$

Where $\Delta G^{\ddagger}$, T and k refer to energy barrier for thermal relaxation (J mol^{-1}), temperature (K) and rate constant (s^{-1}).

$$\ln\left(\frac{k_2}{k_1}\right) = \frac{\Delta G^{\ddagger}}{R} \times \left(\frac{1}{T_1} - \frac{1}{T_2}\right) \qquad \text{Eq. 11}$$

Where k_1 and k_2 refer to the rate constants at elevated temperature (350 K) and room temperature (298 K), respectively; T_1 and T_2 refer to the elevated temperature and room temperature; R refers to ideal gas constant (J K^{-1} mol^{-1}).

Table S4: Kinetic data for the thermal isomerization of the photochromic hydrazone dopants.

	Pentafluoro			*para*-NO_2		
	k / s^{-1}	$\tau_{1/2}$ / year	$\Delta G^{\ddagger}$	k / s^{-1}	$\tau_{1/2}$ / year	$\Delta G^{\ddagger}$
**1**	$6.9 \times 10^{-10} \pm 2.0 \times 10^{-10}$	35 ± 13	30.1 ± 0.20	$1.6 \times 10^{-10} \pm 1.5 \times 10^{-11}$	138 ± 13	30.9 ± 0.10
**2**	$5.3 \times 10^{-10} \pm 4.9 \times 10^{-11}$	41 ± 4	30.2 ± 0.10	$1.5 \times 10^{-10} \pm 8.7 \times 10^{-12}$	147 ± 8	30.9 ± 0.10
**3**	$4.0 \times 10^{-10} \pm 3.7 \times 10^{-12}$	55 ± 0.5	30.4 ± 0.10	---	---	---
**4**	---	---	---	$1.0 \times 10^{-10} \pm 9.8 \times 10^{-12}$	217 ± 21	31.2 ± 0.10

7. HTP (β) and Reflectance of Adaptive Films

Liquid crystalline (LC) samples were prepared by mixing the appropriate amount of LC host 5CB with photochromic dopants **1**–**4**. Mixing was achieved by dissolving the mixture in spectroscopic grade methylene chloride and evaporating under reduced pressure, followed by heating to the isotropic temperature and slow cooling to room temperature. Low concentration mixtures (1.0 mol% or less) were loaded into wedge cells (EHC KCRK-03, -05, -07, -11) at room temperature to give the best alignment. The helical twisting power (β) was calculated using the Grandjean-Cano wedge method.[S6] Briefly, the pitch (p) is proportional to the distance between disclination lines (s) and the angle of the wedge (θ; equation 12). The β value is given by the inverse of pitch multiplied by the concentration (equation 13). To demonstrate the kinetic trapping of the helical assembly at a particular pitch based on the order of sequential irradiation, photochromic samples in 5CB were irradiated with 340 and 442 nm light until the PSS was reached and the β value measured over time to confirm that it does not change. The PSS of the mixture was determined to be the point at which the helical pitch no longer changed with continuous irradiation.

$$p = 2 \cdot s \cdot \tan(\theta) \qquad \text{Eq. 12}$$

$$\beta = \frac{1}{c \cdot p} \qquad \text{Eq. 13}$$

High concentration mixtures (> 2.5 mol%) were loaded into 3 μm (Instec KSRO-03/B507PNSS), 5 μm planar cells (Instec LC3-5.0), and 15 μm homeotropic cells (S100A150uT180) to measure the reflectance of the adaptive films. β and reflectance data for compound **4** were obtained following previous procedures.[S1]

Table S5: Sequential irradiation dependence of the helical pitch and β of compounds **1** and **2** in 5CB.

Wavelength (nm)	**1**			**2**		
	s (μm)	p (μm)	β (μm^{-1})^a	s (μm)	p (μm)	β (μm^{-1})^a
Pristine (*ZZ*)	---	---	---	---	---	---
442 (*ZE*)	240	3.8	51	170	6.2	62
340 (*EZ*)	270	4.2	46	300	11	35
442 (*EE*)	110	1.7	110	130	4.8	81

a β values of compounds **1** and **2** measured in both KCRK03 and KCRK07 wedge cells

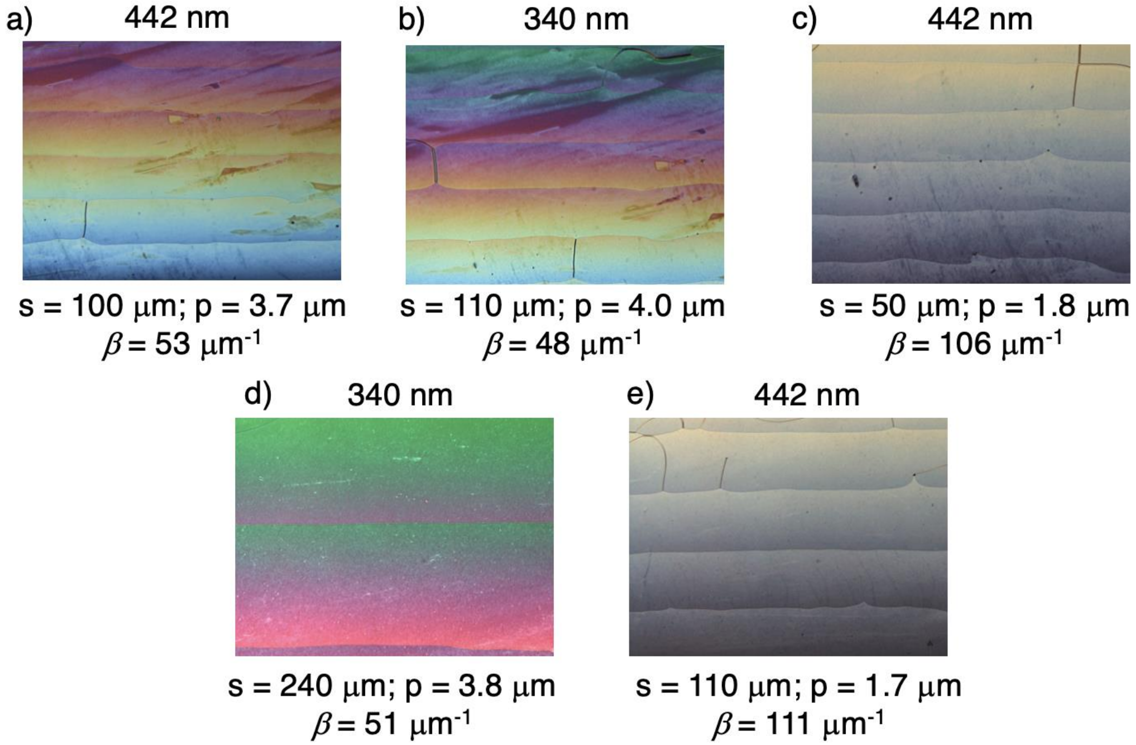

Figure S57: Photomicrographs of compound **1** (0.52 mol%) in 5CB after sequentially irradiating with (a) 442, (b) 340, and (c) 442 light (25x zoom) in a KCRK07 cell and (d) 340 and (e) 442 nm light in a KCRK03 cell.

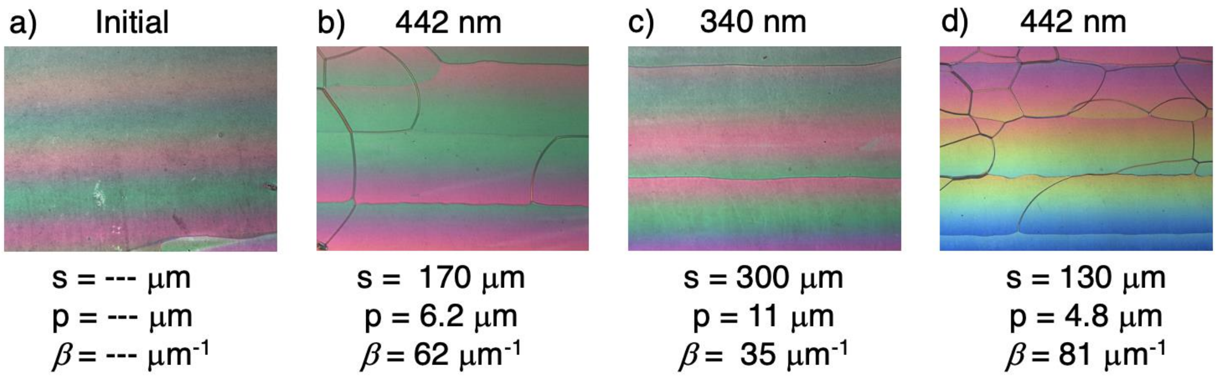

Figure S58: Photomicrographs of compound **2** (0.26 mol%) in 5CB starting with (a) the pristine *ZZ* and after sequentially irradiating with (b) 442, (c) 340, and (d) 442 nm light in a KCRK07 cell.

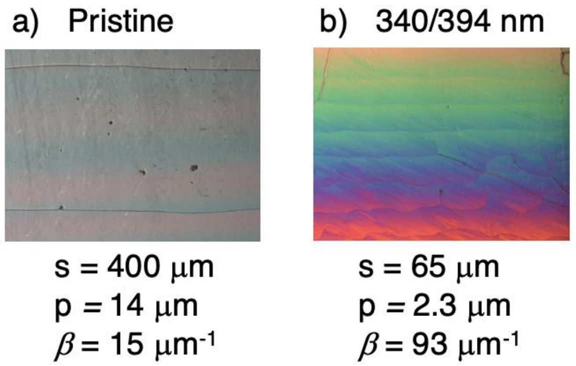

Figure S59: Photomicrographs of compound **3** (0.46 mol%) in 5CB starting with (a) the pristine *ZZ* and after irradiating with (b) 340 nm light in a KCRK07 cell.

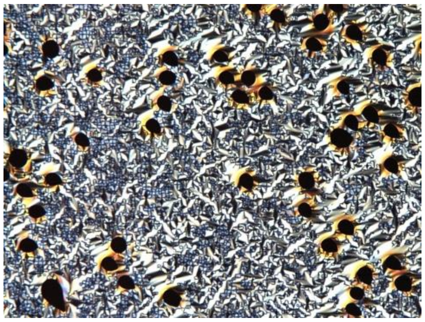

Figure S60: Photomicrograph of compound **3** (3.0 mol%) in 5CB of the pristine state in a LC 3-5 cell.

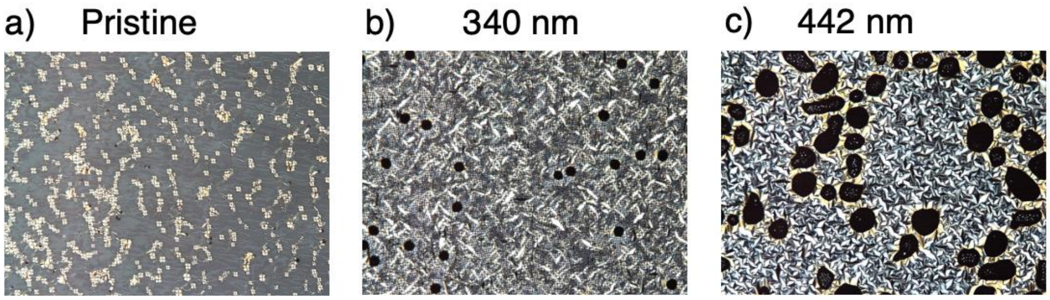

Figure S61: Photomicrographs of a mixture of compounds **3** and **4** (1.5 mol% each) in 5CB after irradiating from the (a) pristine state with (b) 340 and (c) 442 nm light in a LC 3-5 cell. As can be seen the mixing of the equimolar amount of **3** and **4** results in a frustrated texture and only a focal conic phase can be observed after photoirradiation.

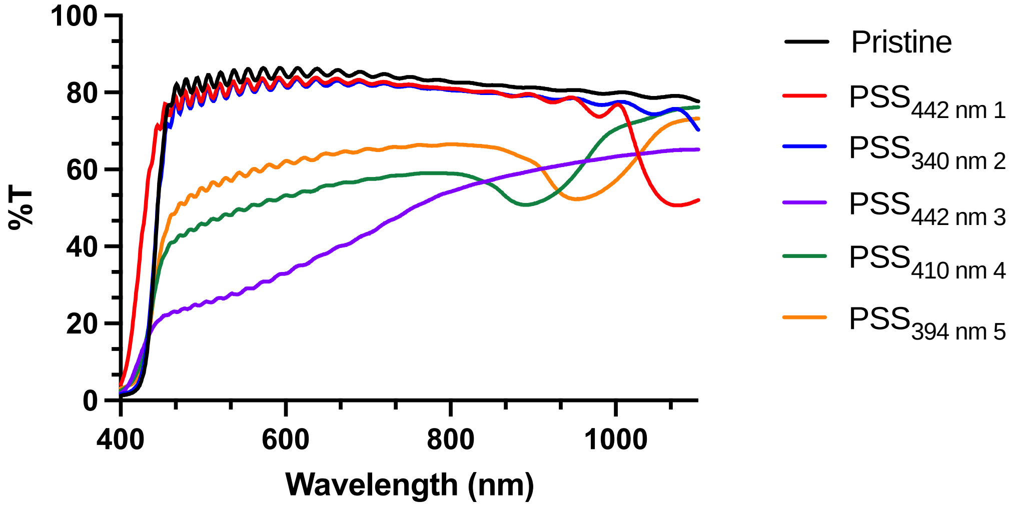

Figure S62: Reflectance modulation starting from the pristine state and sequentially irradiating with 442, 340, 442, 410, and 394 nm light of the LC film containing compound **1** (2.6 mol%) and 5CB in LC 3-5 cell.

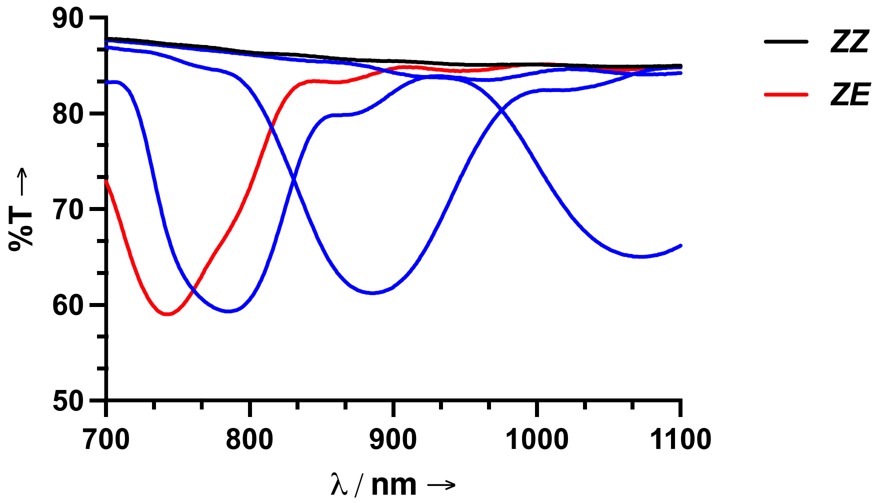

Figure S63: Reflectance modulation starting from the *ZZ* state and sequentially irradiating to *ZE* with 442 light of the LC film containing compound **1** (3.0 mol%) and 5CB in LC KSRO-03/B507PNSS cell.

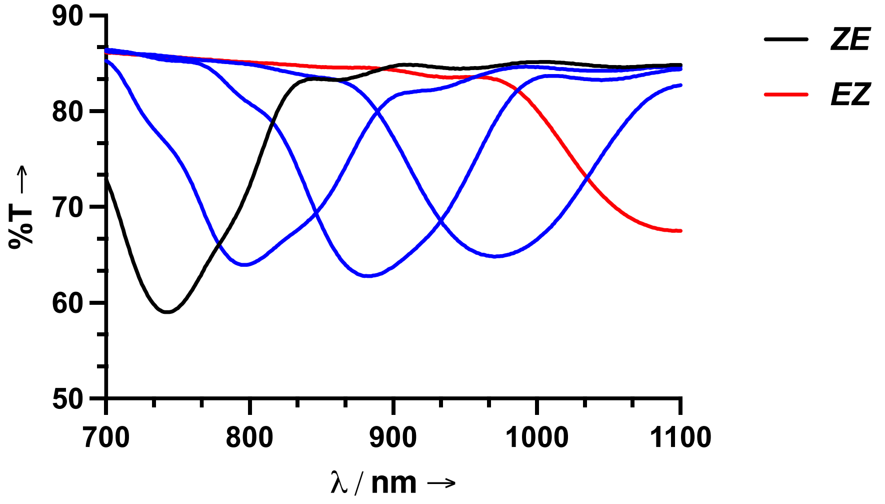

Figure S64: Reflectance modulation starting from the *ZE* state and sequentially irradiating to *EZ* with 340 nm light of the LC film containing compound **1** (3.0 mol%) and 5CB in LC KSRO-03/B507PNSS cell.

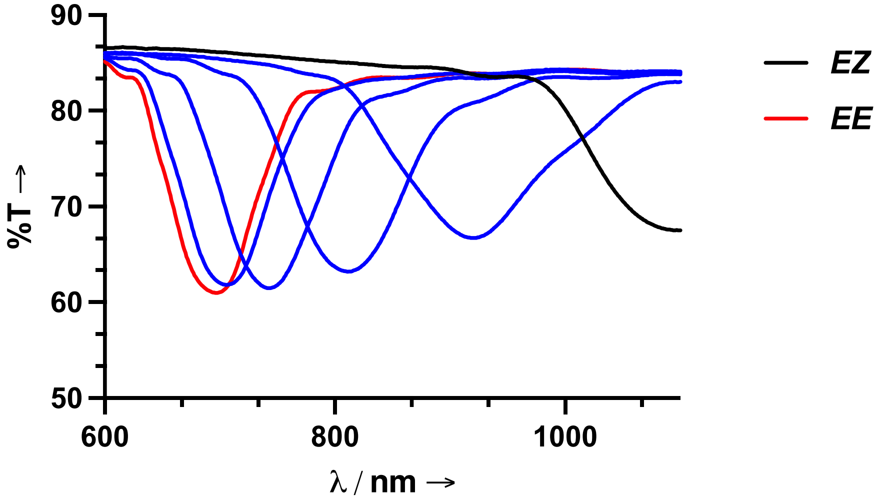

Figure S65: Reflectance modulation starting from the *EZ* state and sequentially irradiating to *EE* with 442 nm light of the LC film containing compound **1** (3.0 mol%) and 5CB in LC KSRO-03/B507PNSS cell.

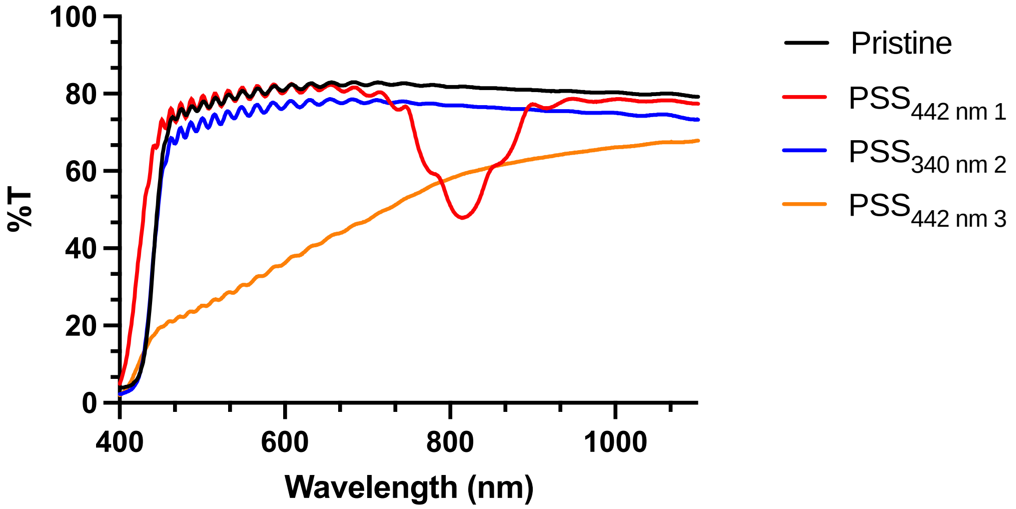

Figure S66: Reflectance modulation starting from the pristine state and sequentially irradiating with 442, 340, and 442 nm light of the LC film containing compound **2** (2.4 mol%) and 5CB in LC 3-5 cell.

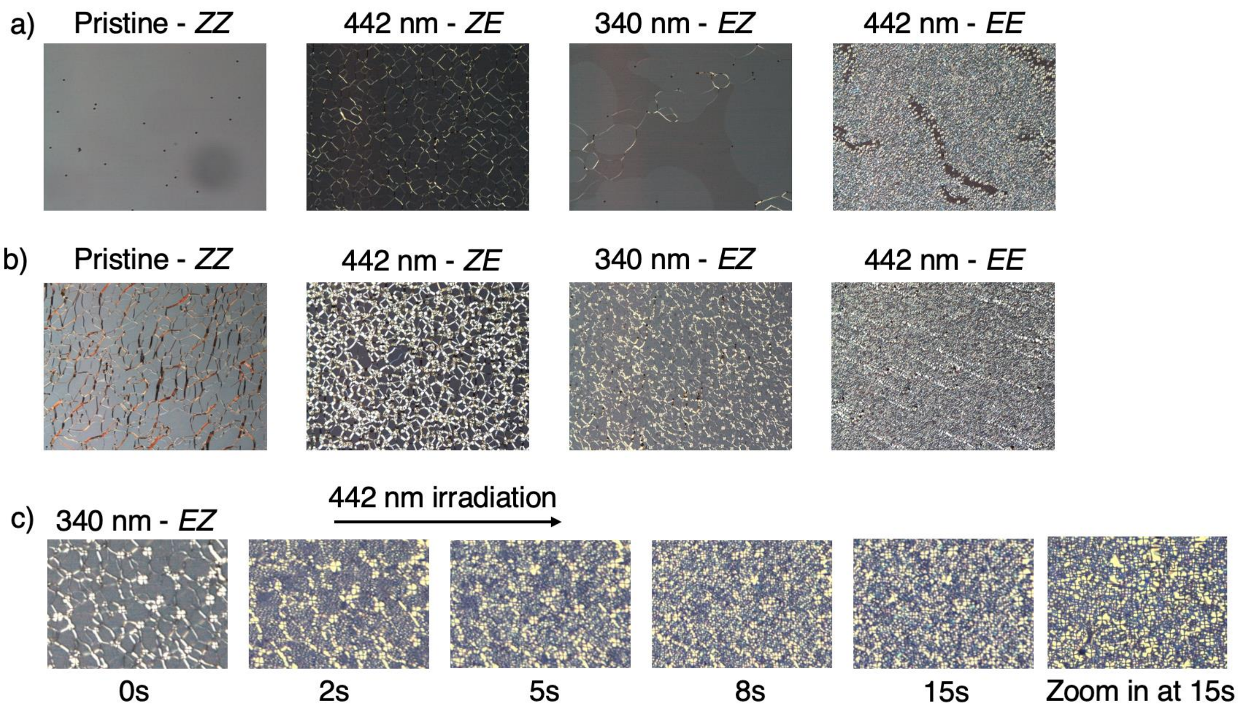

Figure S67: Photomicrographs at the PSS of a) compound **1** (2.6 mol%) and b) compound **2** (2.4 mol%) in 5CB after sequentially irradiating with 442, 340, and 442 nm light in a LC 3-5 cell. c) The focal conic transition that results from *EZ*→*EE* isomerization of **2** upon irradiation with 442 nm light can be observed (10x magnification) at various time stamps until PSS is reached at 15s and the cholesteric texture is no longer observed (25x magnification).

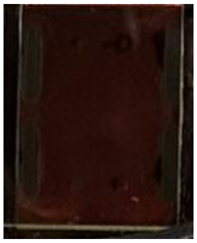

Figure S68: Photomicrographs of compound **1** (2.6 mol%) in 5CB after sequentially irradiating with 340 and 442 nm light in a planar cell with a 3 μm cell gap (KSRO-03/B507PNSS).

8. References

S1. Balamut, B.; Hughes, R. P.; Aprahamian, I. Tuning the Properties of Hydrazone/Isosorbide-Based Switchable Chiral Dopants. *J. Am. Chem. Soc.* **2024**, *146*, 24561–24569.

S2. Qian, H.; Pramanik, S.; Aprahamian, I. Photochromic Hydrazone Switches with Extremely Long Thermal Half-Lives. *J. Am. Chem. Soc.* **2017**, *139*, 9140–9143.

S3. Kuhn, H. J.; Braslavsky, S. E.; Schmidt, R. Chemical Actinometry (IUPAC Technical Report). *Pure and Applied Chemistry* **2004**, *76*, 2105–2146.

S4. Connors, K. A. *Chemical Kinetics: The Study of Reaction Rates in Solution*, Nachdr.; Wiley-VCH: New York, NJ, 1990.

S5. Moran, M. J.; Magrini, M.; Walba, D. M.; Aprahamian, I. Driving a Liquid Crystal Phase Transition Using a Photochromic Hydrazone. *J. Am. Chem. Soc.* **2018**, *140*, 13623–13627.

S6. Podolskyy, D.; Banji, O.; Rudquist, P. Simple Method for Accurate Measurements of the Cholesteric Pitch Using a "Stripe–Wedge" Grandjean–Cano Cell. *Liquid Crystals* **2008**, *35*, 789–791.
